# Effects of various tilt angles on radiation dose and image quality in pediatric head computed tomography: A phantom study

**DOI:** 10.1002/acm2.70177

**Published:** 2025-07-15

**Authors:** Hongrong Xu, Jiawen Zhao, Zhen Xu, Bo Liu, Jinhua Cai

**Affiliations:** ^1^ Department of Radiology National Clinical Research Center for Child Health and Disorders Ministry of Education Key Laboratory of Child Development and Disorders Chongqing Key Laboratory of Structural Birth Defect and Reconstruction Children's Hospital of Chongqing Medical University Chongqing China

**Keywords:** child, computed tomography, head, phantom, radiation

## Abstract

**Background:**

Various methods have been employed to reduce radiation dose and improve image quality in head computed tomography (CT); however, the impact of different head tilt angles on these factors remains underexplored.

**Objective:**

To investigate the effects of different head tilt angles on radiation dose and image quality in head CT.

**Materials and methods:**

A pediatric anthropomorphic head phantom was scanned using dual‐source CT at 18 different tilt angles, repeated 10 times at each angle. Image quality was assessed using mean CT (CT_mean_) attenuation values and image noise at six regions of interest (ROIs), while radiation dose was evaluated using volume CT dose index (CTDI_vol_), size‐specific dose estimate (SSDE), and dose‐length product (DLP). The scan lengths of the eyes and head were also recorded.

**Results:**

CTDI_vol_ and SSDE did not exhibit clear variation patterns with changes in head tilt angles, while DLP was in the lower region between −10° and 5°. Head scan lengths were relatively shorter between −10° and 5°, and eye scan lengths were relatively shorter between −10° and 0°. Except for the image noise of the right middle cranial fossa, no significant differences were found in CT_mean_ values and image noise for the other ROIs between −10° and 0°.

**Conclusion:**

The findings indicated that adjusting the head tilt angle within the range of −10° to 0° reduced both the head and eye scan lengths as well as the radiation dose, while preserving relatively stable image quality.

## 1 INTRODUCTION

Due to the advent of innovative computed tomography (CT) technologies, rapid examination benefits, and widespread installation of CT equipment in hospitals, the number of CT examinations has significantly increased.^1^, ^2^ The frequency of CT examinations in the pediatric population is also increasing.^1^, ^3^, ^4^ Head CT scans have a broad spectrum of indications and are among the most frequently performed CT procedures in the pediatric population. Patients with conditions such as cranial trauma,^6^ intracranial tumors,^7^ cerebral hemorrhage,^8^ or those undergoing shunt surgery for hydrocephalus^9^ may require multiple head CT scans. Under specific circumstances where cumulative exposures occur, there exists a theoretical possibility that radiation doses could approach thresholds for radiosensitive tissues within or near the scanning field.^10^, ^11^

In pediatric populations, susceptibility to x‐ray radiation is inversely proportional to age.^12^ The thyroid and skin of children are more radiosensitive than those of adults, and the risk of radiation‐induced cancer in children is approximately 2–3 times higher than that in adults.^13^ Ocular exposure during head CT scans has been linked to visual impairment and cataractogenesis in high‐dose scenarios; however, current clinical protocols rarely approach deterministic effect thresholds, with routine examinations generally maintaining lens doses below risk‐associated levels even with repeated imaging in most cases.^14^, ^15^

Pediatric head CT scans often face challenges such as uncooperative children, post‐sleep or post‐sedation states, post‐surgery, or tracheal intubation. Hence, different head tilt angles are frequently adapted during head CT examinations in children, complicating the standard positioning of the head.^5^ According to the “as low as reasonably achievable” principle, it is crucial to reduce radiation exposure during medical examinations while maintaining diagnostic image quality, thereby prioritizing patient safety and welfare.^16^ However, few studies have specifically addressed whether different head tilt angles adapted in pediatric head CT scans influence the radiation dose and image quality.

In this study, we employed a pediatric anthropomorphic phantom to assess the impact of different head tilt angles on radiation dose and image quality during head CT examinations. We aimed to investigate a relatively reliable and feasible imaging method to reduce radiation dose and maintain image quality in pediatric head CT scans.

## 2 MATERIALS AND METHODS

### 2.1 Phantom

This phantom study was exempted from institutional review board approval. An anthropomorphic CT phantom (PBU‐70; Kyoto Kagaku Corporation, Kyoto, Japan; height: 110 cm; weight: 20 kg; phantom materials: materials with x‐ray absorption rate equivalent to that of human tissues) was selected to simulate the tissues and organs from the head to feet of a 5‐year‐old child (www.kyotokagaku.com). In this study, only a head phantom was used. The skull component was made from epoxy resin with a density of 1.11 g/cm^3^, while the soft tissue analogue was composed of urethane‐based resin with a density of 1.06 g/cm^3^.

### 2.2 Image data acquisition

All CT scans were performed on a 192‐section dual‐source CT unit (Somatom Force; Siemens Healthcare Sector, Forchheim, Germany) using a head helical mode protocol with Care Dose4D fully activated. The scanning parameters were as follows: collimation, 192 × 0.6 mm; reconstruction thickness, 1 mm; reference mAs, 400 mAs; reference kV, 100 kV; gantry rotation time, 1 s; and pitch, 0.8. The head phantom was positioned in the head holder in the supine orientation. Images were acquired in a caudocranial direction. Scanning extended from the skull base to the top, aiming to minimize the scanning range, while ensuring that the entire head is fully scanned. The table height was adjusted to ensure that the external auditory meatus was centered within the gantry. To minimize radiation exposure to the ocular lenses, the scanning line was aligned parallel to a line connecting the supraorbital ridge and the inner table of the posterior margin of the foramen magnum (reference baseline, [RB]),^5^, ^17^ as recommended by the American Association of Physicists in Medicine. This alignment was achieved by adjusting the head position, as the device did not allow gantry tilting.

**FIGURE 1:**
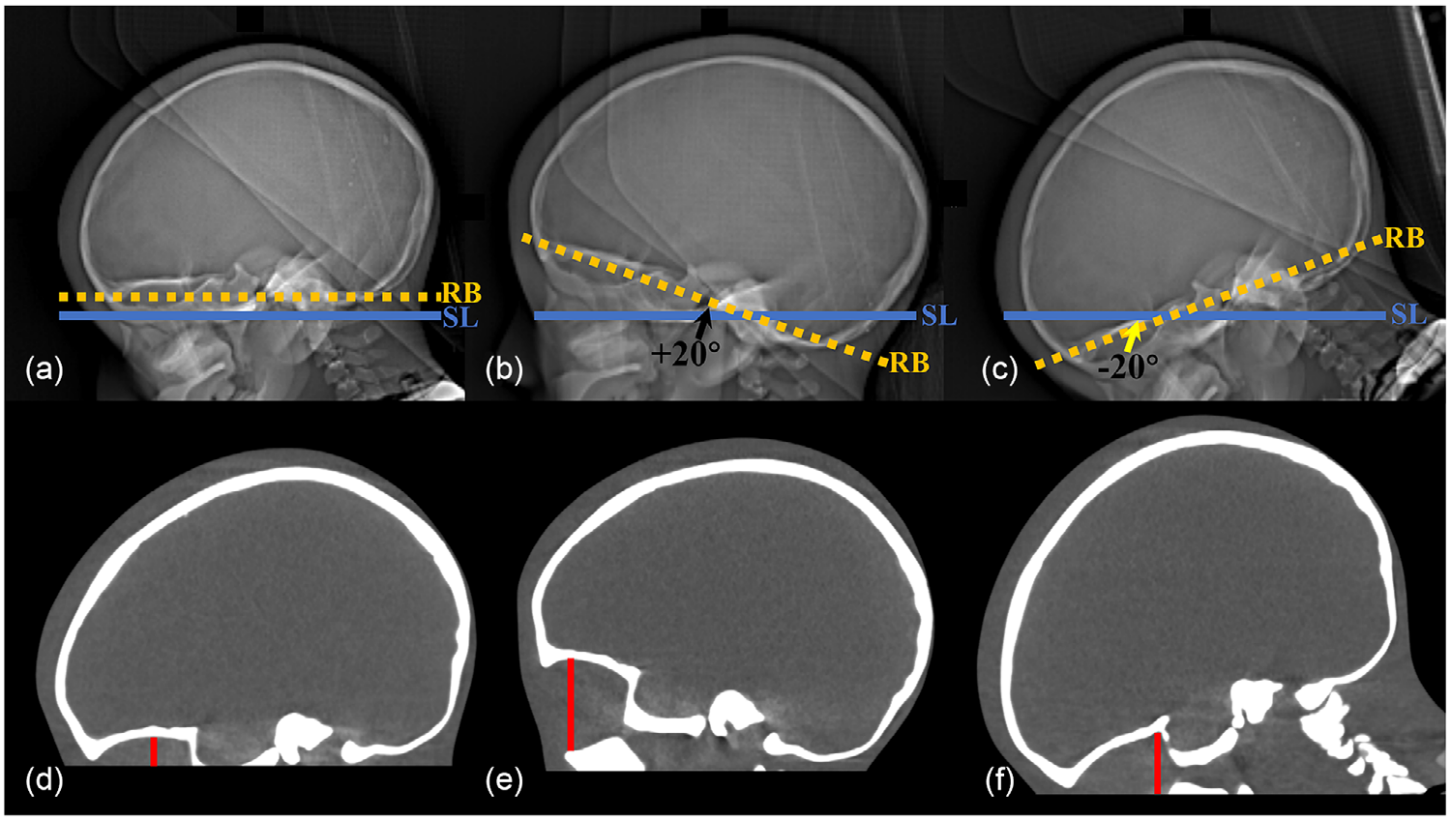
Measurement of head tilt angles and eye scan lengths. (a) 0°: the blue scanning line (SL) aligns with the orange dotted reference baseline ([RB]; supraorbital ridge to posterior foramen magnum inner table); (b) +20°: positive head tilt angle formed by SL and RB (black arrow); (c) −20°: negative head tilt angle (yellow arrow). Sagittal plane measurements of eye scan length (red lines) correspond to: (d) 9 mm at 0°, (e) 37 mm at +20°, and (f) 23.6 mm at −20°.

The head tilt angle was defined as the acute angle between the scanning line and the RB. The angle measurement function was utilized to determine the tilt angle on the scout image. In the standardized position (Figure 1a), the scanning line aligned parallel to the RB, establishing a 0° tilt angle. A positive tilt angle occurred when the chin was elevated away from the chest, causing upward deviation of the RB relative to the scanning line (Figure 1b). Conversely, lowering the chin toward the chest produced a negative tilt angle, with the RB deviating downward (Figure 1c). Head tilt angles ranged from −30° to +55°, divided into 18 groups at 5° intervals. Ten consecutive re‐scans were performed per group using identical acquisition parameters to validate intra‐group consistency and measurement reliability.

Images were reconstructed using the three levels of advanced modelled iterative reconstruction, with a reconstruction kernel of Hr40, and the source images were reconstructed as contiguous axial images with a 1‐mm slice thickness. Volume CT dose index(CTDI_vol_) and dose‐length product (DLP) were recorded from the dose report sheet, with CTDI_vol_ data specific to a 16‐cm diameter dosimetry phantom.

Head scanning length was determined by subtracting the final image localization information from that of the first image in each group. The maximum scanning length of the eyes was measured on the sagittal plane (Figure 1d–f).

On the central slice image encompassing all brain structures in each group, an elliptical region of interest (ROI) was delineated to obtain the area of region of interest (A_ROI_) and average CT (CT_ave_) value within this area to calculate the size‐specific dose estimate (SSDE).^18^, ^19^

The water equivalent diameter (*Dw*) was calculated using Equation (1):

(1) $$Dw=2\sqrt{\left(\frac{{\mathrm{CT}}_{\mathrm{ave}}}{1000}+1\right)\frac{A_{\mathrm{ROI}}}{\pi }}$$

The conversion factor (*f* ^16^) was determined using Equation (2):

(2) $$f^{16}=\alpha e^{-\beta \times Dw}$$

where:

α = 1.9852 [absorbed dose to tissue (mGy)/CTDI_vol_ (mGy)]
β = 0.0486 (cm^−1^)
*e* = the base of the natural logarithm

SSDE was calculated using Equation (3):

(3) $${\mathrm{SSDE}}_{Dw}=f^{16}\times {\mathrm{CTDI}}_{\mathrm{vol}}$$

### 2.3 Image analysis

**FIGURE 2:**
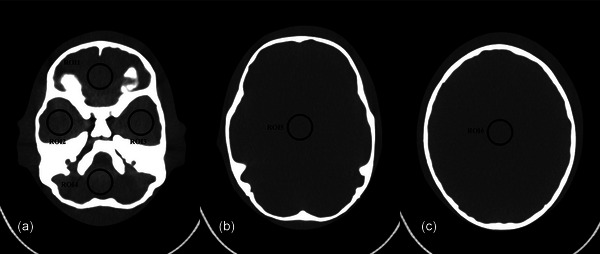
ROIs for quantitative analysis of image quality. ROI 1: Anterior cranial fossa; ROI 2: Right middle cranial fossa; ROI 3: Left middle cranial fossa; ROI 4: Posterior cranial fossa; ROI 5: Central basal ganglia region; ROI 6: Central corpus callosum trunk. CT, computed tomography; ROI, region of interest.

We identified six ROIs that were particularly sensitive to image quality or critical for clinical observation. ROI 1 was located within the anterior cranial fossa; ROI 2 in the right middle cranial fossa; ROI 3 in the left middle cranial fossa; ROI 4 within the posterior cranial fossa; ROI 5 was centrally located in the imaging plane where the basal ganglia was anticipated; and ROI 6 was centrally located in the imaging plane where the trunk of the corpus callosum was anticipated (Figure 2). The image noise for each ROI was represented by the standard deviation of CT value fluctuations. Image quality was assessed based on the variability of the mean CT (CT_mean_) attenuation values and image noise across the six ROIs, and all 18 datasets were analyzed. Multiplanar reconstruction techniques were used to maintain consistent ROI positioning across different scanning groups, with each ROI measuring approximately 200 mm^2^. Image analysis was conducted using an AW Volumeshare 5 workstation (version 4.6; General Electric, Milwaukee, WI, USA).

### 2.4 Statistical analysis

Data were recorded and analyzed using Statistical Product and Service Solutions (version 26, IBM Corporation, Armonk, NY, USA). Continuous numerical data are summarized using the mean ± standard deviation. Scatter plots were used to represent the specific locations and distributions of data points, and the lines connecting the points to describe the trend of change. The statistical analysis was conducted as follows: Normality was assessed using the Shapiro‐Wilk test, and homogeneity of variance was evaluated via Levene's test. For data meeting both normality and homogeneity of variance assumptions, a one‐way analysis of variance (ANOVA) was performed to examine group differences, followed by LSD (Least Significant Difference) post‐hoc multiple comparisons. When the assumption of homogeneity of variance was violated, Welch's ANOVA was applied, and Games‐Howell post‐hoc tests were used for pairwise comparisons. Statistical significance was set at *p* < 0.05.

## 3 RESULTS

Table 1 presents the statistical results for CTDI_vol_, SSDE, DLP, head scan length, and eye scan length at different head tilt angles.

**TABLE 1:** CTDI_vol_, SSDE, DLP, head scan length, and eye scan length variation with different angles.

Angle (°)	CTDI_vol_ (mGy)	SSDE (mGy)	DLP (mGy×cm)	Head scan length(mm)	Eye scan length(mm)
−30	23.48 ± 0.21	21.60 ± 0.19	421.41 ± 3.78	148.00	26.00
−25	22.46 ± 0.26	20.66 ± 0.24	403.01 ± 4.89	148.00	25.40
−20	22.32 ± 0.15	20.47 ± 0.14	387.02 ± 2.65	142.00	23.60
−15	22.33 ± 0.16	20.40 ± 0.15	370.45 ± 2.77	135.00	18.40
−10	22.52 ± 0.18	20.56 ± 0.16	357.78 ± 2.89	127.00	11.90
−5	22.64 ± 0.17	20.72 ± 0.15	349.60 ± 2.67	123.00	9.10
0	22.42 ± 0.34	20.33 ± 0.31	351.46 ± 3.55	124.00	9.00
5	22.45 ± 0.23	20.32 ± 0.21	354.53 ± 3.73	127.00	21.90
10	23.20 ± 0.12	20.85 ± 0.11	372.05 ± 2.03	129.00	29.80
15	22.69 ± 0.18	20.31 ± 0.16	364.98 ± 2.89	130.00	36.00
20	22.19 ± 0.12	19.85 ± 0.10	364.70 ± 1.95	133.00	37.00
25	22.55 ± 0.14	20.21 ± 0.12	377.45 ± 2.33	136.00	37.00
30	23.34 ± 0.13	20.85 ± 0.12	396.57 ± 2.27	139.00	37.00
35	22.69 ± 0.05	20.05 ± 0.05	391.19 ± 0.93	141.00	37.00
40	23.86 ± 0.18	21.01 ± 0.16	422.03 ± 3.17	145.00	37.00
45	24.21 ± 0.11	21.05 ± 0.09	441.49 ± 1.99	151.00	37.00
50	24.63 ± 0.18	21.35 ± 0.16	456.49 ± 3.20	154.00	37.00
55	24.83 ± 0.23	21.27 ± 0.19	460.23 ± 4.14	154.00	37.00

Abbreviations: CTDI_vol_, computed tomography dose index volume; DLP, dose‐length product.; SSDE, size‐specific dose estimate.

### 3.1 CTDI_vol_, SSDE, and DLP variation with head tilt angle

**FIGURE 3:**
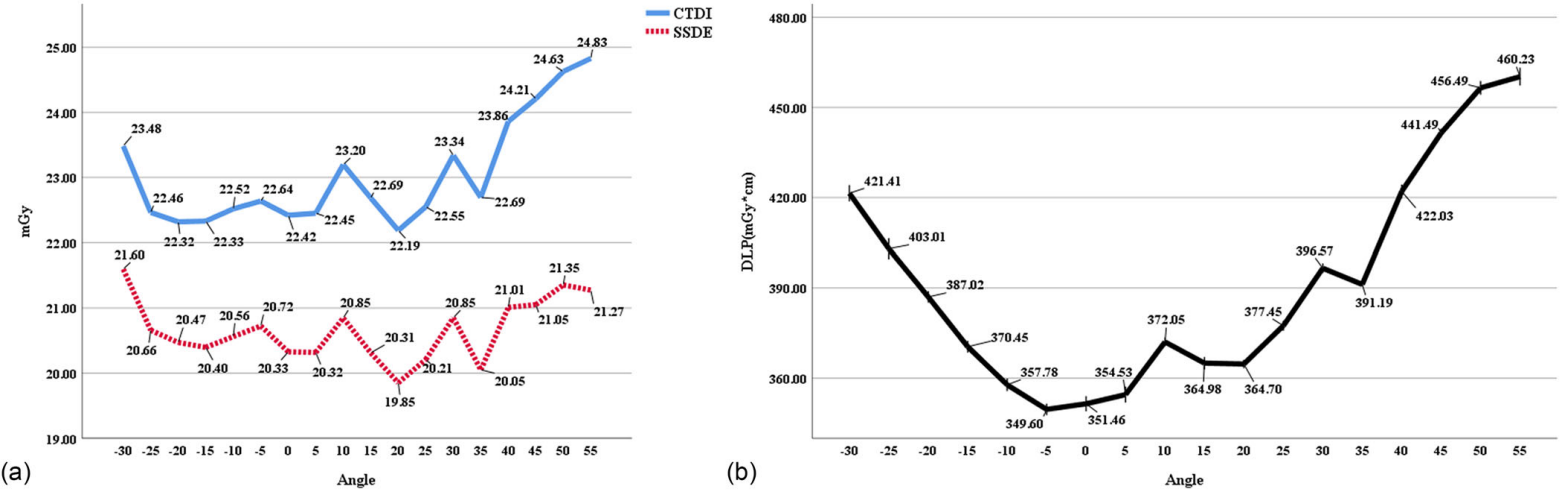
Radiation dose variation versus head tilt angles. (a) CTDI_vol_ and SSDE; (b) DLP. CTDI_vol_, volume computed tomography dose index; SSDE, size‐specific dose estimate; DLP, dose‐length product.

The minimum average CTDI_vol_ value was observed at 20° (22.19 ± 0.12 mGy); however, this value was not significantly different from the values at −25°, −20°, −15°, 0°, and 5° (*p* = 0.333, 0.737, 0.704, 0.796, 0.244, respectively) (Table S1). The minimum average SSDE value was also observed at 20° (19.85 ± 0.10 mGy). Notably, the distribution of the average values of CTDI_vol_ and SSDE across different groups was relatively consistent. The average CTDI_vol_ values were mainly between 22 and 23 mGy, while the average SSDE values were mainly between 20 and 21 mGy, ranging from −25° to 35° (Figure 3a).

The minimum average DLP value was observed at −5° (349.60 ± 2.67 mGy×cm); however, this value was not significantly different from the average value at 0° and 5° (*p* = 0.993, 0.161, respectively) (Table S1). The DLP curve relating to head tilt angle changes showed a relatively flat, low‐value region between −10° and 5°. Outside this region, the remaining angles had larger average values (Figure 3b).

### 3.2 Head and eye scan length variation with head tilt angle

**FIGURE 4:**
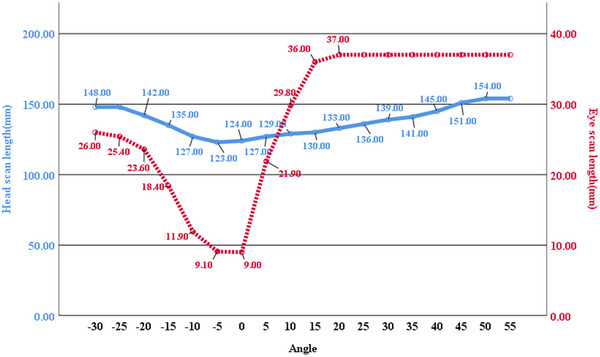
Scan lengths variation versus head tilt angles. Solid blue line represents head scan length, red dotted line represents eye scan length.

The shortest head scan length was 123 mm at −5° and 124 mm at 0°. The shortest eye scan length was 9 mm at 0° and 9.1 mm at −5°. Head and eye scan lengths were relatively shorter within the −10°–15° and −10°–0° ranges, respectively. Outside these ranges, both head and eye scan lengths were relatively longer (Figure 4). Eye scan lengths of 37 mm indicated complete eye scanning.

### 3.3 CT_mean_ value and image noise variation with head tilt angle

**FIGURE 5:**
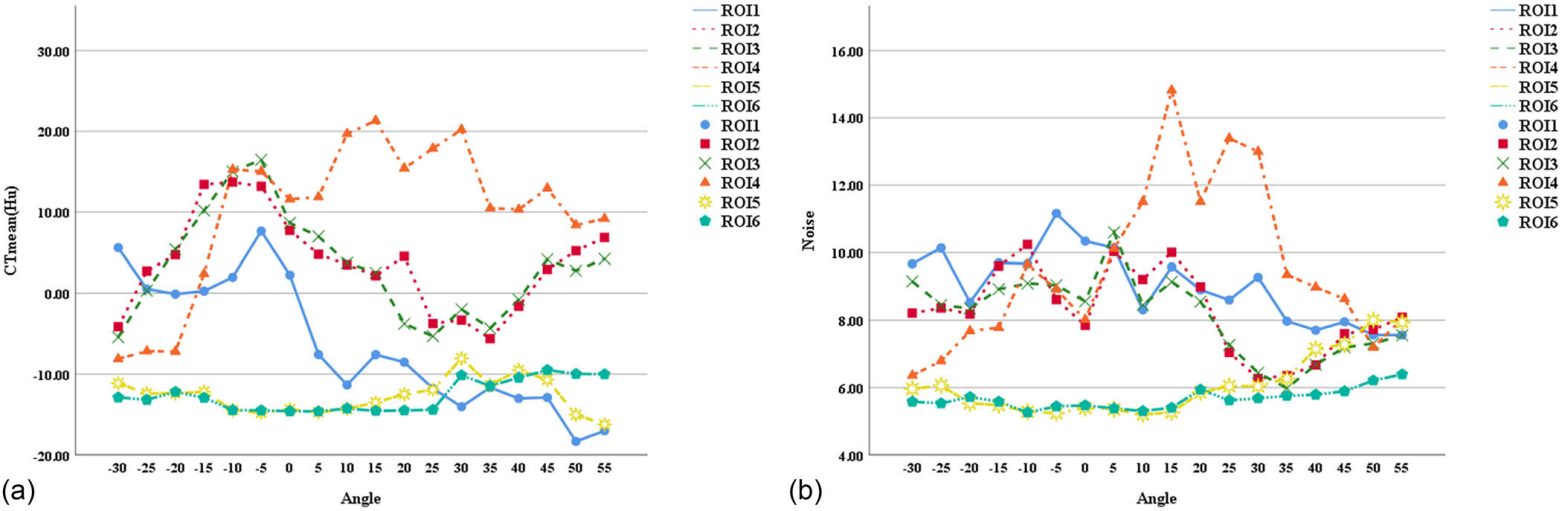
CT_mean_ and image noise variation versus head tilt angles. (a) CT_mean_; (b) image noise. Color‐ and shape‐coded symbols denote different ROIs. CT_mean_, mean computed tomography attenuation value; ROI, region of interest.

**FIGURE 6:**
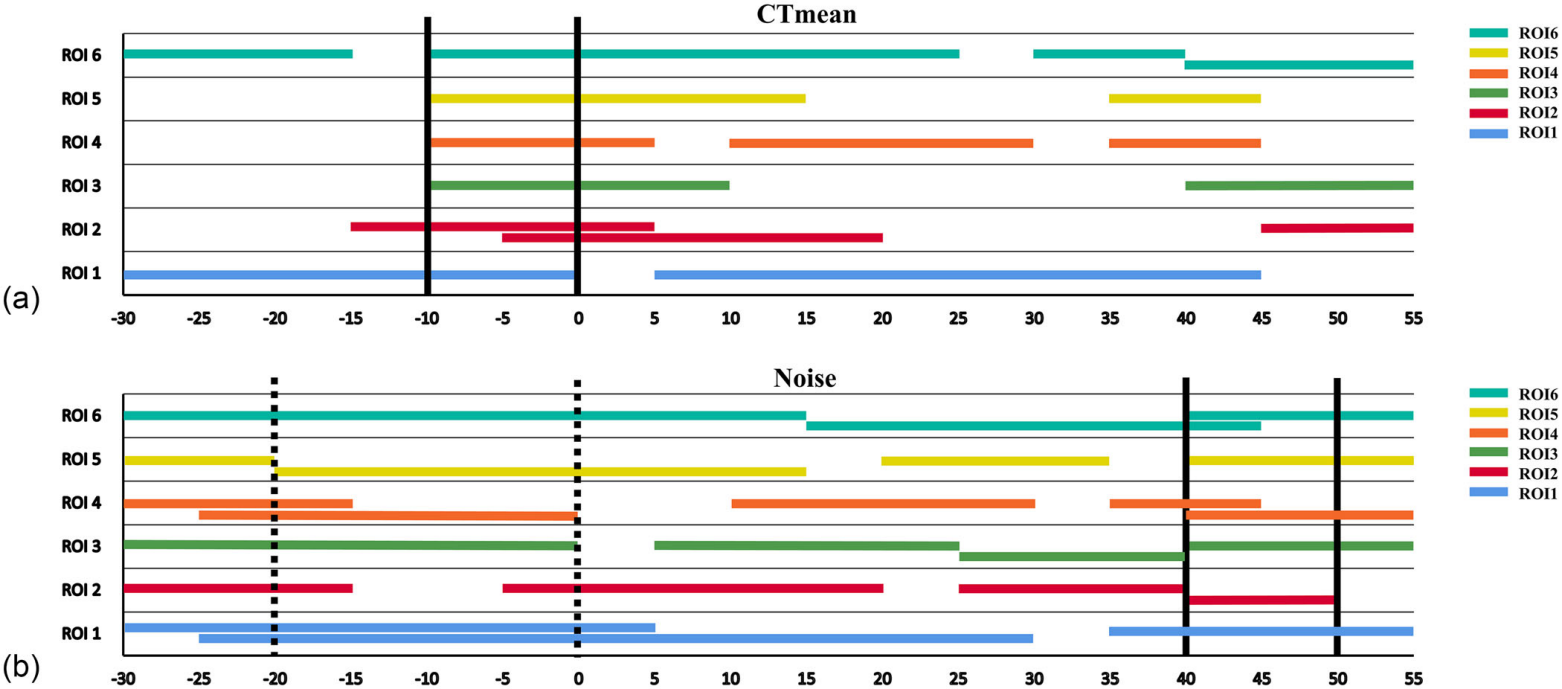
Range of head tilt angles with no statistically significant differences. Color‐coded horizontal lines indicate head tilt angle ranges with no statistically significant differences for individual ROI. (a) CT_mean_: the two solid black vertical lines indicate the angular range (−10°–0°) where differences in CT_mean_ across all six ROIs were not statistically significant. (b) image noise: the two solid black vertical lines indicate the angular range (40°–50°) with non‐significant noise differences for all ROIs, while the two dotted black vertical lines indicate the angular range (−20°–0°) with non‐significant differences for ROIs 1, 3–6 (except ROI 2). CT_mean_, mean computed tomography attenuation value; ROI, region of interest.

The CT_mean_ values and image noise at ROIs 5 and 6 changed minimally with the variation in head tilt angles, while the other four ROIs showed more variations (Figure 5a,b). However, within certain angular ranges, differences in CT_mean_ values and image noise caused by changing head tilt angles were not significant (Table S1). In the six ROIs, the differences in CT_mean_ values were not significant within the −10°–0° range (Figure 6a); the differences in image noise were not significant within the 40°–50° range. From −20° to 0°, image noise differences for ROIs 1,3–6 (except ROI 2) were not significant (Figure 6b).

## 4 DISCUSSION

Few studies have specifically addressed whether different head tilt angles adapted in pediatric head CT examinations influence the radiation dose and image quality. In this study, we used a pediatric anthropomorphic head phantom to assess the impact of radiation dose and image quality with different head tilt angles. In some devices, the gantry cannot be tilted to adjust the scan angle, as described in this study; therefore, only the head posture was modified.

We found that the CTDI_vol_, SSDE, DLP, and head and eye scan lengths of the pediatric phantom varied with head tilt angles.

The shortest eye scan length was observed when the scanning line was parallel to the RB. A positive head tilt is not advisable in head CT scans because a slight increase in the positive tilt angle resulted in a sharp increase in the length of the eye being directly irradiated, significantly increasing the radiation dose received by the eyes. Due to the over‐scanning effect in helical CT scans,^20^ the actual scan length was longer than the values measured when the eyes were positioned at the edge of the head CT scanning field. The use of adaptive dose‐shielding technology can reduce the over‐scanning range and radiation dose at the edge of the scanning volume.^21^, ^22^

The head tilt angle also induced variations in head scan length, with excessive tilting, whether downward or upward, leading to an increase in length. Within the angular range of −10° to 15°, the head scan length remained relatively shorter, varying between 123 and 130 mm. Outside this range, the maximum head scan length reached 154 mm, showing a 31‐mm (25.20%) increase compared to the shortest length. An increased scan length significantly increases the radiation dose and prolongs the scanning duration, thereby increasing the risk of motion artifacts.

All average CTDI_vol_ and DLP values of head CT scans at various tilt angles in this study were lower than the average values of head CT scans for children aged 1–5 years in a national survey in China (39.3 ± 17.2 mGy and 551.1 ± 255 mGy×cm, respectively).^1^ This may likely be because some medical institutions in this survey used adult parameters to scan children and did not adjust the scanning parameters according to age or body size, increasing radiation dose.^1^

ROIs are located at the base of the skull, where the bony structures are numerous and the density of different tissues varies significantly, making these ROIs susceptible to beam hardening artifacts and partial volume averaging effects.^23^, ^24^ At different head tilt angles, the path of the x‐ray beam passing through the ROI to reach the detector changes. The tissues and structures along this changed path vary significantly on the slice of the skull base, leading to shifts in CT values. Despite variations in image noise across all ROIs, numerical changes in most ROIs were not significant. This can likely be attributed to the use of automatic tube current modulation, which aims to maintain image noise at a level predetermined by operators, ensuring consistent image quality.^25^, ^26^ These findings highlight the importance of consistent head positioning during multiple head CT scans, particularly in paediatric patients. Inconsistent head positioning across multiple scans could result in variations in CT values and potentially affect diagnostic accuracy.

Choosing a fixed angle, such as 0° in this study, can expose the eyes to the shortest direct scan length from the x‐ray beam, thereby keeping the radiation dose low. However, setting a precise head tilt angle is challenging for both pediatric patients and radiological technologists. The exact angle for minimal radiation dose may vary slightly depending on the skull shape and size of each child.^27^ A relatively fixed range of head tilt angles can allow some space for children to adjust to a comfortable scanning position, ensuring examination success, which is particularly important for pediatric patients. Radiologists do not need to meticulously adjust the patient's head tilt angle to a precise position; it is only necessary to maintain a slight downward tilt of the head within a limited range. This improves efficiency and reduces radiation dose in pediatric head CT scans.

To date, only a few studies have provided data on the length of direct eye exposure during head CT scans.^14^ This study explored this issue and found that the scanning length of the head varied with different head tilt angles and proposed a method for reducing radiation dose.

Using a pediatric anthropomorphic phantom for head CT scans in this study demonstrated that completely avoiding x‐ray exposure to the eyes was not feasible. According to conclusions drawn by other researchers, the use of shields for eye protection is not recommended,^17^, ^28^ although some researchers consider it beneficial.^29^, ^30^ Shield placement is complex and requires precise placement after obtaining the scout image. Shields can induce imaging artifacts, cause discomfort, and potentially lead to cross‐infection, with the added challenge of ensuring accurate placement.^31^ Maintaining an unaltered head position throughout the procedure is particularly challenging for pediatric patients. Some studies have indicated that combining gantry and head tilt adjustments can avoid or minimize direct x‐ray beam exposure to the eyes.^10^, ^17^ Organ‐based tube current modulation techniques can reduce radiation dose to sensitive organs such as lenses, albeit potentially increasing the radiation dose to the back side of the sensitive organ and image noise.^29^, ^32^, ^33^

Several studies have addressed strategies to lower radiation doses during head CT scans, including precise patient positioning, adjusting the table height to center the head within the bore,^34^ employing automatic tube current modulation techniques,^33^ selecting appropriate tube voltage based on patient size,^21^ narrowing the scan range, utilizing innovative reconstruction algorithms,^35^ applying artificial intelligence to optimize scanning parameters,^36^ implementing paediatric‐specific scanning protocols^6^, ^37^ and monitoring dose reference levels.^38^, ^39^ Not all these technologies are available on specific CT units; however, accurately combining feasible methods can substantially reduce radiation doses in head CT scans. Meticulous positioning of the patient and minimizing scanning length to exclude sensitive organs from the radiation field rely solely on the expertise of the radiology team and can be implemented on most CT units.

This study had some limitations. First, it was conducted using a single pediatric anthropomorphic phantom. Second, the shape and size of the head in the phantom were fixed, which means that shape and size variations of the patient's head may affect the accuracy of the radiation dose and specific angle descriptions. Future studies will require more data from pediatric patients with head CT scans to validate these findings. Third, this study only utilized one CT unit, which implies that different vendors or different CT unit scanning parameters and methods could lead to discrepancies in the assessment of radiation dose and image quality. Future studies should compare the differences in radiation doses and image quality that may result from the use of CT units of different types and vendors.

In conclusion, head position during CT scans can affect radiation exposure to sensitive organs such as the eyes. Adjusting the head tilt angle to a specific range (−10°–0°) reduced both the head and eye scan lengths as well as the radiation dose, while preserving relatively stable image quality.

## AUTHOR CONTRIBUTIONS

**Hongrong Xu**: Conceptualization; methodology; writing—original draft; data curation; investigation. **Jiawen Zhao**: Data curation; formal analysis; investigation; resources. **Zhen Xu**: Investigation; data curation. **Bo Liu**: Methodology; resources; supervision; validation; visualization. **Jinhua Cai**: Writing—reviewing & editing; projection administration; supervision; visualization.

## CONFLICT OF INTEREST STATEMENT

The authors declare no conflicts of interest.

## ETHICAL STATEMENT

This phantom study was exempted from institutional review board approval.

## Supporting information



Supporting Information

## Data Availability

The raw datasets supporting the findings of this study have been deposited in the public repository Mendeley Data (Version 1). The data are openly accessible at: doi:https://doi.org/10.17632/dg668r9fxx.1
