# Supplementary material for: Effects of various tilt angles on radiation dose and image quality in pediatric head computed tomography: A phantom study
Source: J Appl Clin Med Phys. 2025 Jul 15;26(7):e70177. doi: 10.1002/acm2.70177 (PMC12260256; doi:10.1002/acm2.70177)
Supplement: Supplementary file 1 — Supporting Information [file ACM2-26-e70177-s001.docx]

S1 Table. P-values of Welch ANOVA Analysis under different head tilt angles

|  | Angle | -30 | -25 | -20 | -15 | -10 | -5 | 0 | 5 | 10 | 15 | 20 | 25 | 30 | 35 | 40 | 45 | 50 | 55 |
| --- | --- | --- | --- | --- | --- | --- | --- | --- | --- | --- | --- | --- | --- | --- | --- | --- | --- | --- | --- |
| CTDI_vol_ | -30 | NA | 0.000^▲^ | 0.000^▲^ | 0.000^▲^ | 0.000^▲^ | 0.000^▲^ | 0.000^▲^ | 0.000^▲^ | 0.103 | 0.000^▲^ | 0.000^▲^ | 0.000^▲^ | 0.899 | 0.000^▲^ | 0.030^▲^ | 0.000^▲^ | 0.000^▲^ | 0.000^▲^ |
|  | -25 | 0.000^▲^ | NA | 0.980 | 0.992 | 1.000 | 0.926 | 1.000 | 1.000 | 0.000^▲^ | 0.745 | 0.333 | 1.000 | 0.000^▲^ | 0.486 | 0.000^▲^ | 0.000^▲^ | 0.000^▲^ | 0.000^▲^ |
|  | -20 | 0.000^▲^ | 0.980 | NA | 1.000 | 0.460 | 0.023^▲^ | 1.000 | 0.977 | 0.000^▲^ | 0.010^▲^ | 0.737 | 0.113 | 0.000^▲^ | 0.001^▲^ | 0.000^▲^ | 0.000^▲^ | 0.000^▲^ | 0.000^▲^ |
|  | -15 | 0.000^▲^ | 0.992 | 1.000 | NA | 0.598 | 0.042^▲^ | 1.000 | 0.992 | 0.000^▲^ | 0.017^▲^ | 0.704 | 0.199 | 0.000^▲^ | 0.002^▲^ | 0.000^▲^ | 0.000^▲^ | 0.000^▲^ | 0.000^▲^ |
|  | -10 | 0.000^▲^ | 1.000 | 0.460 | 0.598 | NA | 0.973 | 1.000 | 1.000 | 0.000^▲^ | 0.799 | 0.013^▲^ | 1.000 | 0.000^▲^ | 0.348 | 0.000^▲^ | 0.000^▲^ | 0.000^▲^ | 0.000^▲^ |
|  | -5 | 0.000^▲^ | 0.926 | 0.023^▲^ | 0.042^▲^ | 0.973 | NA | 0.900 | 0.815 | 0.000^▲^ | 1.000 | 0.000^▲^ | 0.995 | 0.000^▲^ | 1.000 | 0.000^▲^ | 0.000^▲^ | 0.000^▲^ | 0.000^▲^ |
|  | 0 | 0.000^▲^ | 1.000 | 1.000 | 1.000 | 1.000 | 0.900 | NA | 1.000 | 0.002^▲^ | 0.739 | 0.796 | 0.998 | 0.000^▲^ | 0.560 | 0.000^▲^ | 0.000^▲^ | 0.000^▲^ | 0.000^▲^ |
|  | 5 | 0.000^▲^ | 1.000 | 0.977 | 0.992 | 1.000 | 0.815 | 1.000 | NA | 0.000^▲^ | 0.560 | 0.244 | 0.998 | 0.000^▲^ | 0.274 | 0.000^▲^ | 0.000^▲^ | 0.000^▲^ | 0.000^▲^ |
|  | 10 | 0.103 | 0.000^▲^ | 0.000^▲^ | 0.000^▲^ | 0.000^▲^ | 0.000^▲^ | 0.002^▲^ | 0.000^▲^ | NA | 0.000^▲^ | 0.000^▲^ | 0.000^▲^ | 0.561 | 0.000^▲^ | 0.000^▲^ | 0.000^▲^ | 0.000^▲^ | 0.000^▲^ |
|  | 15 | 0.000^▲^ | 0.745 | 0.010^▲^ | 0.017^▲^ | 0.799 | 1.000 | 0.739 | 0.560 | 0.000^▲^ | NA | 0.000^▲^ | 0.887 | 0.000^▲^ | 1.000 | 0.000^▲^ | 0.000^▲^ | 0.000^▲^ | 0.000^▲^ |
|  | 20 | 0.000^▲^ | 0.333 | 0.737 | 0.704 | 0.013^▲^ | 0.000^▲^ | 0.796 | 0.244 | 0.000^▲^ | 0.000^▲^ | NA | 0.001^▲^ | 0.000^▲^ | 0.000^▲^ | 0.000^▲^ | 0.000^▲^ | 0.000^▲^ | 0.000^▲^ |
|  | 25 | 0.000^▲^ | 1.000 | 0.113 | 0.199 | 1.000 | 0.995 | 0.998 | 0.998 | 0.000^▲^ | 0.887 | 0.001^▲^ | NA | 0.000^▲^ | 0.297 | 0.000^▲^ | 0.000^▲^ | 0.000^▲^ | 0.000^▲^ |
|  | 30 | 0.899 | 0.000^▲^ | 0.000^▲^ | 0.000^▲^ | 0.000^▲^ | 0.000^▲^ | 0.000^▲^ | 0.000^▲^ | 0.561 | 0.000^▲^ | 0.000^▲^ | 0.000^▲^ | NA | 0.000^▲^ | 0.000^▲^ | 0.000^▲^ | 0.000^▲^ | 0.000^▲^ |
|  | 35 | 0.000^▲^ | 0.486 | 0.001^▲^ | 0.002^▲^ | 0.348 | 1.000 | 0.560 | 0.274 | 0.000^▲^ | 1.000 | 0.000^▲^ | 0.297 | 0.000^▲^ | NA | 0.000^▲^ | 0.000^▲^ | 0.000^▲^ | 0.000^▲^ |
|  | 40 | 0.030^▲^ | 0.000^▲^ | 0.000^▲^ | 0.000^▲^ | 0.000^▲^ | 0.000^▲^ | 0.000^▲^ | 0.000^▲^ | 0.000^▲^ | 0.000^▲^ | 0.000^▲^ | 0.000^▲^ | 0.000^▲^ | 0.000^▲^ | NA | 0.009^▲^ | 0.000^▲^ | 0.000^▲^ |
|  | 45 | 0.000^▲^ | 0.000^▲^ | 0.000^▲^ | 0.000^▲^ | 0.000^▲^ | 0.000^▲^ | 0.000^▲^ | 0.000^▲^ | 0.000^▲^ | 0.000^▲^ | 0.000^▲^ | 0.000^▲^ | 0.000^▲^ | 0.000^▲^ | 0.009^▲^ | NA | 0.001^▲^ | 0.000^▲^ |
|  | 50 | 0.000^▲^ | 0.000^▲^ | 0.000^▲^ | 0.000^▲^ | 0.000^▲^ | 0.000^▲^ | 0.000^▲^ | 0.000^▲^ | 0.000^▲^ | 0.000^▲^ | 0.000^▲^ | 0.000^▲^ | 0.000^▲^ | 0.000^▲^ | 0.000^▲^ | 0.001^▲^ | NA | 0.721 |
|  | 55 | 0.000^▲^ | 0.000^▲^ | 0.000^▲^ | 0.000^▲^ | 0.000^▲^ | 0.000^▲^ | 0.000^▲^ | 0.000^▲^ | 0.000^▲^ | 0.000^▲^ | 0.000^▲^ | 0.000^▲^ | 0.000^▲^ | 0.000^▲^ | 0.000^▲^ | 0.000^▲^ | 0.721 | NA |
| SSDE | -30 | NA | 0.000^▲^ | 0.000^▲^ | 0.000^▲^ | 0.000^▲^ | 0.000^▲^ | 0.000^▲^ | 0.000^▲^ | 0.000^▲^ | 0.000^▲^ | 0.000^▲^ | 0.000^▲^ | 0.000^▲^ | 0.000^▲^ | 0.000^▲^ | 0.000^▲^ | 0.288 | 0.088 |
|  | -25 | 0.000^▲^ | NA | 0.779 | 0.362 | 0.999 | 1.000 | 0.462 | 0.182 | 0.744 | 0.102 | 0.000^▲^ | 0.011^▲^ | 0.727 | 0.001^▲^ | 0.093 | 0.030^▲^ | 0.000^▲^ | 0.001^▲^ |
|  | -20 | 0.000^▲^ | 0.779 | NA | 0.998 | 0.992 | 0.070 | 0.989 | 0.873 | 0.000^▲^ | 0.652 | 0.000^▲^ | 0.021^▲^ | 0.000^▲^ | 0.000^▲^ | 0.000^▲^ | 0.000^▲^ | 0.000^▲^ | 0.000^▲^ |
|  | -15 | 0.000^▲^ | 0.362 | 0.998 | NA | 0.646 | 0.011^▲^ | 1.000 | 1.000 | 0.000^▲^ | 0.997 | 0.000^▲^ | 0.265 | 0.000^▲^ | 0.002^▲^ | 0.000^▲^ | 0.000^▲^ | 0.000^▲^ | 0.000^▲^ |
|  | -10 | 0.000^▲^ | 0.999 | 0.992 | 0.646 | NA | 0.692 | 0.766 | 0.357 | 0.023^▲^ | 0.155 | 0.000^▲^ | 0.004^▲^ | 0.023^▲^ | 0.000^▲^ | 0.001^▲^ | 0.000^▲^ | 0.000^▲^ | 0.000^▲^ |
|  | -5 | 0.000^▲^ | 1.000 | 0.070 | 0.011^▲^ | 0.692 | NA | 0.124 | 0.011^▲^ | 0.837 | 0.002^▲^ | 0.000^▲^ | 0.000^▲^ | 0.821 | 0.000^▲^ | 0.051 | 0.004^▲^ | 0.000^▲^ | 0.000^▲^ |
|  | 0 | 0.000^▲^ | 0.462 | 0.989 | 1.000 | 0.766 | 0.124 | NA | 1.000 | 0.019^▲^ | 1.000 | 0.035^▲^ | 0.998 | 0.018^▲^ | 0.428 | 0.002^▲^ | 0.001^▲^ | 0.000^▲^ | 0.000^▲^ |
|  | 5 | 0.000^▲^ | 0.182 | 0.873 | 1.000 | 0.357 | 0.011^▲^ | 1.000 | NA | 0.001^▲^ | 1.000 | 0.002^▲^ | 0.986 | 0.001^▲^ | 0.112 | 0.000^▲^ | 0.000^▲^ | 0.000^▲^ | 0.000^▲^ |
|  | 10 | 0.000^▲^ | 0.744 | 0.000^▲^ | 0.000^▲^ | 0.023^▲^ | 0.837 | 0.019^▲^ | 0.001^▲^ | NA | 0.000^▲^ | 0.000^▲^ | 0.000^▲^ | 1.000 | 0.000^▲^ | 0.489 | 0.026^▲^ | 0.000^▲^ | 0.002^▲^ |
|  | 15 | 0.000^▲^ | 0.102 | 0.652 | 0.997 | 0.155 | 0.002^▲^ | 1.000 | 1.000 | 0.000^▲^ | NA | 0.000^▲^ | 0.966 | 0.000^▲^ | 0.030^▲^ | 0.000^▲^ | 0.000^▲^ | 0.000^▲^ | 0.000^▲^ |
|  | 20 | 0.000^▲^ | 0.000^▲^ | 0.000^▲^ | 0.000^▲^ | 0.000^▲^ | 0.000^▲^ | 0.035^▲^ | 0.002^▲^ | 0.000^▲^ | 0.000^▲^ | NA | 0.000^▲^ | 0.000^▲^ | 0.007^▲^ | 0.000^▲^ | 0.000^▲^ | 0.000^▲^ | 0.000^▲^ |
|  | 25 | 0.000^▲^ | 0.011^▲^ | 0.021^▲^ | 0.265 | 0.004^▲^ | 0.000^▲^ | 0.998 | 0.986 | 0.000^▲^ | 0.966 | 0.000^▲^ | NA | 0.000^▲^ | 0.108 | 0.000^▲^ | 0.000^▲^ | 0.000^▲^ | 0.000^▲^ |
|  | 30 | 0.000^▲^ | 0.727 | 0.000^▲^ | 0.000^▲^ | 0.023^▲^ | 0.821 | 0.018^▲^ | 0.001^▲^ | 1.000 | 0.000^▲^ | 0.000^▲^ | 0.000^▲^ | NA | 0.000^▲^ | 0.559 | 0.042^▲^ | 0.000^▲^ | 0.002^▲^ |
|  | 35 | 0.000^▲^ | 0.001^▲^ | 0.000^▲^ | 0.002^▲^ | 0.000^▲^ | 0.000^▲^ | 0.428 | 0.112 | 0.000^▲^ | 0.030^▲^ | 0.007^▲^ | 0.108 | 0.000^▲^ | NA | 0.000^▲^ | 0.000^▲^ | 0.000^▲^ | 0.000^▲^ |
|  | 40 | 0.000^▲^ | 0.093 | 0.000^▲^ | 0.000^▲^ | 0.001^▲^ | 0.051 | 0.002^▲^ | 0.000^▲^ | 0.489 | 0.000^▲^ | 0.000^▲^ | 0.000^▲^ | 0.559 | 0.000^▲^ | NA | 1.000 | 0.013^▲^ | 0.186 |
|  | 45 | 0.000^▲^ | 0.030^▲^ | 0.000^▲^ | 0.000^▲^ | 0.000^▲^ | 0.004^▲^ | 0.001^▲^ | 0.000^▲^ | 0.026^▲^ | 0.000^▲^ | 0.000^▲^ | 0.000^▲^ | 0.042^▲^ | 0.000^▲^ | 1.000 | NA | 0.012^▲^ | 0.229 |
|  | 50 | 0.288 | 0.000^▲^ | 0.000^▲^ | 0.000^▲^ | 0.000^▲^ | 0.000^▲^ | 0.000^▲^ | 0.000^▲^ | 0.000^▲^ | 0.000^▲^ | 0.000^▲^ | 0.000^▲^ | 0.000^▲^ | 0.000^▲^ | 0.013^▲^ | 0.012^▲^ | NA | 1.000 |
|  | 55 | 0.088 | 0.001^▲^ | 0.000^▲^ | 0.000^▲^ | 0.000^▲^ | 0.000^▲^ | 0.000^▲^ | 0.000^▲^ | 0.002^▲^ | 0.000^▲^ | 0.000^▲^ | 0.000^▲^ | 0.002^▲^ | 0.000^▲^ | 0.186 | 0.229 | 1.000 | NA |
| DLP | -30 | NA | 0.000^▲^ | 0.000^▲^ | 0.000^▲^ | 0.000^▲^ | 0.000^▲^ | 0.000^▲^ | 0.000^▲^ | 0.000^▲^ | 0.000^▲^ | 0.000^▲^ | 0.000^▲^ | 0.000^▲^ | 0.000^▲^ | 1.000 | 0.000^▲^ | 0.000^▲^ | 0.000^▲^ |
|  | -25 | 0.000^▲^ | NA | 0.000^▲^ | 0.000^▲^ | 0.000^▲^ | 0.000^▲^ | 0.000^▲^ | 0.000^▲^ | 0.000^▲^ | 0.000^▲^ | 0.000^▲^ | 0.000^▲^ | 0.105 | 0.001^▲^ | 0.000^▲^ | 0.000^▲^ | 0.000^▲^ | 0.000^▲^ |
|  | -20 | 0.000^▲^ | 0.000^▲^ | NA | 0.000^▲^ | 0.000^▲^ | 0.000^▲^ | 0.000^▲^ | 0.000^▲^ | 0.000^▲^ | 0.000^▲^ | 0.000^▲^ | 0.000^▲^ | 0.000^▲^ | 0.031^▲^ | 0.000^▲^ | 0.000^▲^ | 0.000^▲^ | 0.000^▲^ |
|  | -15 | 0.000^▲^ | 0.000^▲^ | 0.000^▲^ | NA | 0.000^▲^ | 0.000^▲^ | 0.000^▲^ | 0.000^▲^ | 0.981 | 0.028^▲^ | 0.005^▲^ | 0.001^▲^ | 0.000^▲^ | 0.000^▲^ | 0.000^▲^ | 0.000^▲^ | 0.000^▲^ | 0.000^▲^ |
|  | -10 | 0.000^▲^ | 0.000^▲^ | 0.000^▲^ | 0.000^▲^ | NA | 0.000^▲^ | 0.027^▲^ | 0.743 | 0.000^▲^ | 0.002^▲^ | 0.001^▲^ | 0.000^▲^ | 0.000^▲^ | 0.000^▲^ | 0.000^▲^ | 0.000^▲^ | 0.000^▲^ | 0.000^▲^ |
|  | -5 | 0.000^▲^ | 0.000^▲^ | 0.000^▲^ | 0.000^▲^ | 0.000^▲^ | NA | 0.993 | 0.161 | 0.000^▲^ | 0.000^▲^ | 0.000^▲^ | 0.000^▲^ | 0.000^▲^ | 0.000^▲^ | 0.000^▲^ | 0.000^▲^ | 0.000^▲^ | 0.000^▲^ |
|  | 0 | 0.000^▲^ | 0.000^▲^ | 0.000^▲^ | 0.000^▲^ | 0.027^▲^ | 0.993 | NA | 0.884 | 0.000^▲^ | 0.000^▲^ | 0.000^▲^ | 0.000^▲^ | 0.000^▲^ | 0.000^▲^ | 0.000^▲^ | 0.000^▲^ | 0.000^▲^ | 0.000^▲^ |
|  | 5 | 0.000^▲^ | 0.000^▲^ | 0.000^▲^ | 0.000^▲^ | 0.743 | 0.161 | 0.884 | NA | 0.000^▲^ | 0.000^▲^ | 0.000^▲^ | 0.000^▲^ | 0.000^▲^ | 0.000^▲^ | 0.000^▲^ | 0.000^▲^ | 0.000^▲^ | 0.000^▲^ |
|  | 10 | 0.000^▲^ | 0.000^▲^ | 0.000^▲^ | 0.981 | 0.000^▲^ | 0.000^▲^ | 0.000^▲^ | 0.000^▲^ | NA | 0.001^▲^ | 0.000^▲^ | 0.003^▲^ | 0.000^▲^ | 0.000^▲^ | 0.000^▲^ | 0.000^▲^ | 0.000^▲^ | 0.000^▲^ |
|  | 15 | 0.000^▲^ | 0.000^▲^ | 0.000^▲^ | 0.028^▲^ | 0.002^▲^ | 0.000^▲^ | 0.000^▲^ | 0.000^▲^ | 0.001^▲^ | NA | 1.000 | 0.000^▲^ | 0.000^▲^ | 0.000^▲^ | 0.000^▲^ | 0.000^▲^ | 0.000^▲^ | 0.000^▲^ |
|  | 20 | 0.000^▲^ | 0.000^▲^ | 0.000^▲^ | 0.005^▲^ | 0.001^▲^ | 0.000^▲^ | 0.000^▲^ | 0.000^▲^ | 0.000^▲^ | 1.000 | NA | 0.000^▲^ | 0.000^▲^ | 0.000^▲^ | 0.000^▲^ | 0.000^▲^ | 0.000^▲^ | 0.000^▲^ |
|  | 25 | 0.000^▲^ | 0.000^▲^ | 0.000^▲^ | 0.001^▲^ | 0.000^▲^ | 0.000^▲^ | 0.000^▲^ | 0.000^▲^ | 0.003^▲^ | 0.000^▲^ | 0.000^▲^ | NA | 0.000^▲^ | 0.000^▲^ | 0.000^▲^ | 0.000^▲^ | 0.000^▲^ | 0.000^▲^ |
|  | 30 | 0.000^▲^ | 0.105 | 0.000^▲^ | 0.000^▲^ | 0.000^▲^ | 0.000^▲^ | 0.000^▲^ | 0.000^▲^ | 0.000^▲^ | 0.000^▲^ | 0.000^▲^ | 0.000^▲^ | NA | 0.001^▲^ | 0.000^▲^ | 0.000^▲^ | 0.000^▲^ | 0.000^▲^ |
|  | 35 | 0.000^▲^ | 0.001^▲^ | 0.031^▲^ | 0.000^▲^ | 0.000^▲^ | 0.000^▲^ | 0.000^▲^ | 0.000^▲^ | 0.000^▲^ | 0.000^▲^ | 0.000^▲^ | 0.000^▲^ | 0.001^▲^ | NA | 0.000^▲^ | 0.000^▲^ | 0.000^▲^ | 0.000^▲^ |
|  | 40 | 1.000 | 0.000^▲^ | 0.000^▲^ | 0.000^▲^ | 0.000^▲^ | 0.000^▲^ | 0.000^▲^ | 0.000^▲^ | 0.000^▲^ | 0.000^▲^ | 0.000^▲^ | 0.000^▲^ | 0.000^▲^ | 0.000^▲^ | NA | 0.000^▲^ | 0.000^▲^ | 0.000^▲^ |
|  | 45 | 0.000^▲^ | 0.000^▲^ | 0.000^▲^ | 0.000^▲^ | 0.000^▲^ | 0.000^▲^ | 0.000^▲^ | 0.000^▲^ | 0.000^▲^ | 0.000^▲^ | 0.000^▲^ | 0.000^▲^ | 0.000^▲^ | 0.000^▲^ | 0.000^▲^ | NA | 0.000^▲^ | 0.000^▲^ |
|  | 50 | 0.000^▲^ | 0.000^▲^ | 0.000^▲^ | 0.000^▲^ | 0.000^▲^ | 0.000^▲^ | 0.000^▲^ | 0.000^▲^ | 0.000^▲^ | 0.000^▲^ | 0.000^▲^ | 0.000^▲^ | 0.000^▲^ | 0.000^▲^ | 0.000^▲^ | 0.000^▲^ | NA | 0.698 |
|  | 55 | 0.000^▲^ | 0.000^▲^ | 0.000^▲^ | 0.000^▲^ | 0.000^▲^ | 0.000^▲^ | 0.000^▲^ | 0.000^▲^ | 0.000^▲^ | 0.000^▲^ | 0.000^▲^ | 0.000^▲^ | 0.000^▲^ | 0.000^▲^ | 0.000^▲^ | 0.000^▲^ | 0.698 | NA |
| ROI1 CT_mean_ | -30 | NA | 0.455 | 0.148 | 0.699 | 0.883 | 1.000 | 0.994 | 0.010^▲^ | 0.000^▲^ | 0.205 | 0.002^▲^ | 0.000^▲^ | 0.000^▲^ | 0.000^▲^ | 0.000^▲^ | 0.000^▲^ | 0.000^▲^ | 0.000^▲^ |
|  | -25 | 0.455 | NA | 1.000 | 1.000 | 1.000 | 0.473 | 1.000 | 0.297 | 0.005^▲^ | 0.812 | 0.102 | 0.000^▲^ | 0.000^▲^ | 0.000^▲^ | 0.000^▲^ | 0.000^▲^ | 0.000^▲^ | 0.000^▲^ |
|  | -20 | 0.148 | 1.000 | NA | 1.000 | 0.999 | 0.287 | 1.000 | 0.333 | 0.004^▲^ | 0.855 | 0.112 | 0.000^▲^ | 0.000^▲^ | 0.000^▲^ | 0.000^▲^ | 0.000^▲^ | 0.000^▲^ | 0.000^▲^ |
|  | -15 | 0.699 | 1.000 | 1.000 | NA | 1.000 | 0.596 | 1.000 | 0.509 | 0.027^▲^ | 0.891 | 0.256 | 0.010^▲^ | 0.002^▲^ | 0.010^▲^ | 0.004^▲^ | 0.004^▲^ | 0.000^▲^ | 0.000^▲^ |
|  | -10 | 0.883 | 1.000 | 0.999 | 1.000 | NA | 0.785 | 1.000 | 0.134 | 0.001^▲^ | 0.629 | 0.037^▲^ | 0.000^▲^ | 0.000^▲^ | 0.000^▲^ | 0.000^▲^ | 0.000^▲^ | 0.000^▲^ | 0.000^▲^ |
|  | -5 | 1.000 | 0.473 | 0.287 | 0.596 | 0.785 | NA | 0.952 | 0.011^▲^ | 0.000^▲^ | 0.143 | 0.003^▲^ | 0.000^▲^ | 0.000^▲^ | 0.000^▲^ | 0.000^▲^ | 0.000^▲^ | 0.000^▲^ | 0.000^▲^ |
|  | 0 | 0.994 | 1.000 | 1.000 | 1.000 | 1.000 | 0.952 | NA | 0.288 | 0.017^▲^ | 0.714 | 0.134 | 0.009^▲^ | 0.002^▲^ | 0.010^▲^ | 0.004^▲^ | 0.005^▲^ | 0.001^▲^ | 0.000^▲^ |
|  | 5 | 0.010^▲^ | 0.297 | 0.333 | 0.509 | 0.134 | 0.011^▲^ | 0.288 | NA | 0.992 | 1.000 | 1.000 | 0.942 | 0.547 | 0.913 | 0.699 | 0.734 | 0.039^▲^ | 0.108 |
|  | 10 | 0.000^▲^ | 0.005^▲^ | 0.004^▲^ | 0.027^▲^ | 0.001^▲^ | 0.000^▲^ | 0.017^▲^ | 0.992 | NA | 1.000 | 0.999 | 1.000 | 0.995 | 1.000 | 1.000 | 1.000 | 0.095 | 0.424 |
|  | 15 | 0.205 | 0.812 | 0.855 | 0.891 | 0.629 | 0.143 | 0.714 | 1.000 | 1.000 | NA | 1.000 | 0.999 | 0.945 | 0.998 | 0.981 | 0.985 | 0.397 | 0.613 |
|  | 20 | 0.002^▲^ | 0.102 | 0.112 | 0.256 | 0.037^▲^ | 0.003^▲^ | 0.134 | 1.000 | 0.999 | 1.000 | NA | 0.984 | 0.636 | 0.969 | 0.793 | 0.828 | 0.032^▲^ | 0.108 |
|  | 25 | 0.000^▲^ | 0.000^▲^ | 0.000^▲^ | 0.010^▲^ | 0.000^▲^ | 0.000^▲^ | 0.009^▲^ | 0.942 | 1.000 | 0.999 | 0.984 | NA | 0.992 | 1.000 | 1.000 | 1.000 | 0.011^▲^ | 0.215 |
|  | 30 | 0.000^▲^ | 0.000^▲^ | 0.000^▲^ | 0.002^▲^ | 0.000^▲^ | 0.000^▲^ | 0.002^▲^ | 0.547 | 0.995 | 0.945 | 0.636 | 0.992 | NA | 0.932 | 1.000 | 1.000 | 0.215 | 0.930 |
|  | 35 | 0.000^▲^ | 0.000^▲^ | 0.000^▲^ | 0.010^▲^ | 0.000^▲^ | 0.000^▲^ | 0.010^▲^ | 0.913 | 1.000 | 0.998 | 0.969 | 1.000 | 0.932 | NA | 0.996 | 0.999 | 0.000^▲^ | 0.068 |
|  | 40 | 0.000^▲^ | 0.000^▲^ | 0.000^▲^ | 0.004^▲^ | 0.000^▲^ | 0.000^▲^ | 0.004^▲^ | 0.699 | 1.000 | 0.981 | 0.793 | 1.000 | 1.000 | 0.996 | NA | 1.000 | 0.008^▲^ | 0.438 |
|  | 45 | 0.000^▲^ | 0.000^▲^ | 0.000^▲^ | 0.004^▲^ | 0.000^▲^ | 0.000^▲^ | 0.005^▲^ | 0.734 | 1.000 | 0.985 | 0.828 | 1.000 | 1.000 | 0.999 | 1.000 | NA | 0.010^▲^ | 0.423 |
|  | 50 | 0.000^▲^ | 0.000^▲^ | 0.000^▲^ | 0.000^▲^ | 0.000^▲^ | 0.000 | 0.001^▲^ | 0.039^▲^ | 0.095 | 0.397 | 0.032^▲^ | 0.011^▲^ | 0.215 | 0.000^▲^ | 0.008^▲^ | 0.010^▲^ | NA | 0.999 |
|  | 55 | 0.000^▲^ | 0.000^▲^ | 0.000^▲^ | 0.000^▲^ | 0.000^▲^ | 0.000^▲^ | 0.000^▲^ | 0.108 | 0.424 | 0.613 | 0.108 | 0.215 | 0.930 | 0.068 | 0.438 | 0.423 | 0.999 | NA |
| ROI2 CT_mean_ | -30 | NA | 0.006^▲^ | 0.001^▲^ | 0.000^▲^ | 0.000^▲^ | 0.006^▲^ | 0.022^▲^ | 0.067 | 0.006^▲^ | 0.069 | 0.005^▲^ | 1.000 | 1.000 | 0.996 | 0.777 | 0.019^▲^ | 0.000^▲^ | 0.000^▲^ |
|  | -25 | 0.006^▲^ | NA | 0.841 | 0.000^▲^ | 0.002^▲^ | 0.148 | 0.747 | 0.999 | 1.000 | 1.000 | 0.992 | 0.001^▲^ | 0.000^▲^ | 0.000^▲^ | 0.001^▲^ | 1.000 | 0.436 | 0.094 |
|  | -20 | 0.001^▲^ | 0.841 | NA | 0.001^▲^ | 0.009^▲^ | 0.369 | 0.994 | 1.000 | 1.000 | 0.924 | 1.000 | 0.000^▲^ | 0.000^▲^ | 0.000^▲^ | 0.001^▲^ | 0.994 | 1.000 | 0.943 |
|  | -15 | 0.000^▲^ | 0.000^▲^ | 0.001^▲^ | NA | 1.000 | 1.000 | 0.712 | 0.089 | 0.000^▲^ | 0.000^▲^ | 0.005^▲^ | 0.000^▲^ | 0.000^▲^ | 0.000^▲^ | 0.000^▲^ | 0.000^▲^ | 0.002^▲^ | 0.026^▲^ |
|  | -10 | 0.000^▲^ | 0.002^▲^ | 0.009^▲^ | 1.000 | NA | 1.000 | 0.738 | 0.111 | 0.003^▲^ | 0.001^▲^ | 0.016^▲^ | 0.000^▲^ | 0.000^▲^ | 0.000^▲^ | 0.000^▲^ | 0.002^▲^ | 0.014^▲^ | 0.083 |
|  | -5 | 0.006^▲^ | 0.148 | 0.369 | 1.000 | 1.000 | NA | 0.977 | 0.575 | 0.232 | 0.132 | 0.395 | 0.007^▲^ | 0.010^▲^ | 0.004^▲^ | 0.020^▲^ | 0.185 | 0.434 | 0.750 |
|  | 0 | 0.022^▲^ | 0.747 | 0.994 | 0.712 | 0.738 | 0.977 | NA | 1.000 | 0.929 | 0.734 | 0.996 | 0.027^▲^ | 0.035^▲^ | 0.010^▲^ | 0.091 | 0.863 | 0.999 | 1.000 |
|  | 5 | 0.067 | 0.999 | 1.000 | 0.089 | 0.111 | 0.575 | 1.000 | NA | 1.000 | 0.998 | 1.000 | 0.075 | 0.088 | 0.019^▲^ | 0.269 | 1.000 | 1.000 | 1.000 |
|  | 10 | 0.006^▲^ | 1.000 | 1.000 | 0.000^▲^ | 0.003^▲^ | 0.232 | 0.929 | 1.000 | NA | 1.000 | 1.000 | 0.003^▲^ | 0.003^▲^ | 0.000^▲^ | 0.028^▲^ | 1.000 | 0.990 | 0.592 |
|  | 15 | 0.069 | 1.000 | 0.924 | 0.000^▲^ | 0.001^▲^ | 0.132 | 0.734 | 0.998 | 1.000 | NA | 0.991 | 0.058 | 0.054 | 0.004^▲^ | 0.364 | 1.000 | 0.738 | 0.259 |
|  | 20 | 0.005^▲^ | 0.992 | 1.000 | 0.005^▲^ | 0.016^▲^ | 0.395 | 0.996 | 1.000 | 1.000 | 0.991 | NA | 0.004^▲^ | 0.007^▲^ | 0.001^▲^ | 0.037^▲^ | 1.000 | 1.000 | 0.982 |
|  | 25 | 1.000 | 0.001^▲^ | 0.000^▲^ | 0.000^▲^ | 0.000^▲^ | 0.007^▲^ | 0.027^▲^ | 0.075 | 0.003^▲^ | 0.058 | 0.004^▲^ | NA | 1.000 | 0.872 | 0.739 | 0.013^▲^ | 0.000^▲^ | 0.000^▲^ |
|  | 30 | 1.000 | 0.000^▲^ | 0.000^▲^ | 0.000^▲^ | 0.000^▲^ | 0.010^▲^ | 0.035^▲^ | 0.088 | 0.003^▲^ | 0.054 | 0.007^▲^ | 1.000 | NA | 0.040^▲^ | 0.292 | 0.013^▲^ | 0.000^▲^ | 0.000^▲^ |
|  | 35 | 0.996 | 0.000^▲^ | 0.000^▲^ | 0.000^▲^ | 0.000^▲^ | 0.004^▲^ | 0.010^▲^ | 0.019^▲^ | 0.000^▲^ | 0.004^▲^ | 0.001^▲^ | 0.872 | 0.040^▲^ | NA | 0.001^▲^ | 0.001^▲^ | 0.000^▲^ | 0.000^▲^ |
|  | 40 | 0.777 | 0.001^▲^ | 0.001^▲^ | 0.000^▲^ | 0.000^▲^ | 0.020^▲^ | 0.091 | 0.269 | 0.028^▲^ | 0.364 | 0.037^▲^ | 0.739 | 0.292 | 0.001^▲^ | NA | 0.112 | 0.000^▲^ | 0.000^▲^ |
|  | 45 | 0.019^▲^ | 1.000 | 0.994 | 0.000^▲^ | 0.002^▲^ | 0.185 | 0.863 | 1.000 | 1.000 | 1.000 | 1.000 | 0.013^▲^ | 0.013^▲^ | 0.001^▲^ | 0.112 | NA | 0.933 | 0.437 |
|  | 50 | 0.000^▲^ | 0.436 | 1.000 | 0.002^▲^ | 0.014^▲^ | 0.434 | 0.999 | 1.000 | 0.990 | 0.738 | 1.000 | 0.000^▲^ | 0.000^▲^ | 0.000^▲^ | 0.000^▲^ | 0.933 | NA | 0.989 |
|  | 55 | 0.000^▲^ | 0.094 | 0.943 | 0.026^▲^ | 0.083 | 0.750 | 1.000 | 1.000 | 0.592 | 0.259 | 0.982 | 0.000^▲^ | 0.000^▲^ | 0.000^▲^ | 0.000^▲^ | 0.437 | 0.989 | NA |
| ROI3 CT_mean_ | -30 | NA | 0.005^▲^ | 0.000^▲^ | 0.000^▲^ | 0.001^▲^ | 0.003^▲^ | 0.006^▲^ | 0.001^▲^ | 0.001^▲^ | 0.013^▲^ | 0.982 | 1.000 | 0.112 | 0.991 | 0.017^▲^ | 0.032^▲^ | 0.000^▲^ | 0.000^▲^ |
|  | -25 | 0.005^▲^ | NA | 0.003^▲^ | 0.000^▲^ | 0.012^▲^ | 0.026^▲^ | 0.175 | 0.123 | 0.535 | 0.984 | 0.055 | 0.001^▲^ | 0.336 | 0.004^▲^ | 0.991 | 0.875 | 0.288 | 0.018^▲^ |
|  | -20 | 0.000^▲^ | 0.003^▲^ | NA | 0.016^▲^ | 0.151 | 0.200 | 0.985 | 1.000 | 0.994 | 0.872 | 0.000^▲^ | 0.000^▲^ | 0.000^▲^ | 0.000^▲^ | 0.000^▲^ | 1.000 | 0.119 | 0.970 |
|  | -15 | 0.000^▲^ | 0.000^▲^ | 0.016^▲^ | NA | 0.903 | 0.852 | 1.000 | 0.929 | 0.020^▲^ | 0.018^▲^ | 0.000^▲^ | 0.000^▲^ | 0.000^▲^ | 0.000^▲^ | 0.000^▲^ | 0.374 | 0.000^▲^ | 0.001^▲^ |
|  | -10 | 0.001^▲^ | 0.012^▲^ | 0.151 | 0.903 | NA | 1.000 | 0.895 | 0.488 | 0.074 | 0.043^▲^ | 0.002^▲^ | 0.001^▲^ | 0.004^▲^ | 0.002^▲^ | 0.007^▲^ | 0.165 | 0.040^▲^ | 0.083 |
|  | -5 | 0.003^▲^ | 0.026^▲^ | 0.200 | 0.852 | 1.000 | NA | 0.829 | 0.475 | 0.113 | 0.072 | 0.005^▲^ | 0.003^▲^ | 0.011^▲^ | 0.005^▲^ | 0.017^▲^ | 0.189 | 0.069 | 0.124 |
|  | 0 | 0.006^▲^ | 0.175 | 0.985 | 1.000 | 0.895 | 0.829 | NA | 1.000 | 0.857 | 0.673 | 0.016^▲^ | 0.007^▲^ | 0.045^▲^ | 0.013^▲^ | 0.088 | 0.978 | 0.545 | 0.854 |
|  | 5 | 0.001^▲^ | 0.123 | 1.000 | 0.929 | 0.488 | 0.475 | 1.000 | NA | 0.959 | 0.804 | 0.004^▲^ | 0.002^▲^ | 0.018^▲^ | 0.004^▲^ | 0.045^▲^ | 0.999 | 0.582 | 0.950 |
|  | 10 | 0.001^▲^ | 0.535 | 0.994 | 0.020^▲^ | 0.074 | 0.113 | 0.857 | 0.959 | NA | 1.000 | 0.004^▲^ | 0.001^▲^ | 0.029^▲^ | 0.003^▲^ | 0.125 | 1.000 | 1.000 | 1.000 |
|  | 15 | 0.013^▲^ | 0.984 | 0.872 | 0.018^▲^ | 0.043^▲^ | 0.072 | 0.673 | 0.804 | 1.000 | NA | 0.069 | 0.013^▲^ | 0.277 | 0.032^▲^ | 0.676 | 1.000 | 1.000 | 0.997 |
|  | 20 | 0.982 | 0.055 | 0.000^▲^ | 0.000^▲^ | 0.002^▲^ | 0.005^▲^ | 0.016^▲^ | 0.004^▲^ | 0.004^▲^ | 0.069 | NA | 0.904 | 0.739 | 1.000 | 0.161 | 0.103 | 0.000^▲^ | 0.000^▲^ |
|  | 25 | 1.000 | 0.001^▲^ | 0.000^▲^ | 0.000^▲^ | 0.001^▲^ | 0.003^▲^ | 0.007^▲^ | 0.002^▲^ | 0.001^▲^ | 0.013^▲^ | 0.904 | NA | 0.001^▲^ | 0.711 | 0.000^▲^ | 0.033^▲^ | 0.000^▲^ | 0.000^▲^ |
|  | 30 | 0.112 | 0.336 | 0.000^▲^ | 0.000^▲^ | 0.004^▲^ | 0.011^▲^ | 0.045^▲^ | 0.018^▲^ | 0.029^▲^ | 0.277 | 0.739 | 0.001^▲^ | NA | 0.002^▲^ | 0.731 | 0.289 | 0.000^▲^ | 0.000^▲^ |
|  | 35 | 0.991 | 0.004^▲^ | 0.000^▲^ | 0.000^▲^ | 0.002^▲^ | 0.005^▲^ | 0.013^▲^ | 0.004^▲^ | 0.003^▲^ | 0.032^▲^ | 1.000 | 0.711 | 0.002^▲^ | NA | 0.000^▲^ | 0.064 | 0.000^▲^ | 0.000^▲^ |
|  | 40 | 0.017^▲^ | 0.991 | 0.000^▲^ | 0.000^▲^ | 0.007^▲^ | 0.017^▲^ | 0.088 | 0.045^▲^ | 0.125 | 0.676 | 0.161 | 0.000^▲^ | 0.731 | 0.000^▲^ | NA | 0.561 | 0.001^▲^ | 0.000^▲^ |
|  | 45 | 0.032^▲^ | 0.875 | 1.000 | 0.374 | 0.165 | 0.189 | 0.978 | 0.999 | 1.000 | 1.000 | 0.103 | 0.033^▲^ | 0.289 | 0.064 | 0.561 | NA | 1.000 | 1.000 |
|  | 50 | 0.000^▲^ | 0.288 | 0.119 | 0.000^▲^ | 0.040^▲^ | 0.069 | 0.545 | 0.582 | 1.000 | 1.000 | 0.000^▲^ | 0.000^▲^ | 0.000^▲^ | 0.000^▲^ | 0.001^▲^ | 1.000 | NA | 0.550 |
|  | 55 | 0.000^▲^ | 0.018^▲^ | 0.970 | 0.001^▲^ | 0.083 | 0.124 | 0.854 | 0.950 | 1.000 | 0.997 | 0.000^▲^ | 0.000^▲^ | 0.000^▲^ | 0.000^▲^ | 0.000^▲^ | 1.000 | 0.550 | NA |
| ROI4 CT_mean_ | -30 | NA | 1.000 | 1.000 | 0.000^▲^ | 0.000^▲^ | 0.000^▲^ | 0.000^▲^ | 0.000^▲^ | 0.000^▲^ | 0.000 | 0.000^▲^ | 0.000^▲^ | 0.000^▲^ | 0.000^▲^ | 0.000^▲^ | 0.000^▲^ | 0.000^▲^ | 0.000^▲^ |
|  | -25 | 1.000 | NA | 1.000 | 0.001^▲^ | 0.000^▲^ | 0.000^▲^ | 0.000^▲^ | 0.000^▲^ | 0.000^▲^ | 0.000 | 0.000^▲^ | 0.000^▲^ | 0.000^▲^ | 0.000^▲^ | 0.000^▲^ | 0.000^▲^ | 0.000^▲^ | 0.000^▲^ |
|  | -20 | 1.000 | 1.000 | NA | 0.001^▲^ | 0.000^▲^ | 0.000^▲^ | 0.000^▲^ | 0.000^▲^ | 0.000^▲^ | 0.000 | 0.000^▲^ | 0.000^▲^ | 0.000^▲^ | 0.000^▲^ | 0.000^▲^ | 0.000^▲^ | 0.000^▲^ | 0.000^▲^ |
|  | -15 | 0.000^▲^ | 0.001^▲^ | 0.001^▲^ | NA | 0.005^▲^ | 0.007^▲^ | 0.068 | 0.001^▲^ | 0.000^▲^ | 0.000 | 0.000^▲^ | 0.004^▲^ | 0.000^▲^ | 0.002^▲^ | 0.003^▲^ | 0.000^▲^ | 0.021^▲^ | 0.010^▲^ |
|  | -10 | 0.000^▲^ | 0.000^▲^ | 0.000^▲^ | 0.005^▲^ | NA | 1.000 | 0.995 | 0.982 | 0.921 | 0.682 | 1.000 | 1.000 | 0.922 | 0.677 | 0.704 | 0.999 | 0.229 | 0.403 |
|  | -5 | 0.000^▲^ | 0.000^▲^ | 0.000^▲^ | 0.007^▲^ | 1.000 | NA | 0.998 | 0.992 | 0.893 | 0.638 | 1.000 | 1.000 | 0.897 | 0.752 | 0.776 | 1.000 | 0.279 | 0.475 |
|  | 0 | 0.000^▲^ | 0.000^▲^ | 0.000^▲^ | 0.068 | 0.995 | 0.998 | NA | 1.000 | 0.195 | 0.091 | 0.967 | 0.821 | 0.240 | 1.000 | 1.000 | 1.000 | 0.955 | 0.998 |
|  | 5 | 0.000^▲^ | 0.000^▲^ | 0.000^▲^ | 0.001^▲^ | 0.982 | 0.992 | 1.000 | NA | 0.036^▲^ | 0.020 | 0.849 | 0.684 | 0.095 | 0.999 | 0.999 | 1.000 | 0.382 | 0.848 |
|  | 10 | 0.000^▲^ | 0.000^▲^ | 0.000^▲^ | 0.000^▲^ | 0.921 | 0.893 | 0.195 | 0.036^▲^ | NA | 1.000 | 0.760 | 1.000 | 1.000 | 0.005^▲^ | 0.004^▲^ | 0.060 | 0.001^▲^ | 0.001^▲^ |
|  | 15 | 0.000^▲^ | 0.000^▲^ | 0.000^▲^ | 0.000^▲^ | 0.682 | 0.638 | 0.091 | 0.020^▲^ | 1.000 | NA | 0.434 | 0.998 | 1.000 | 0.005^▲^ | 0.004^▲^ | 0.035^▲^ | 0.001^▲^ | 0.002^▲^ |
|  | 20 | 0.000^▲^ | 0.000^▲^ | 0.000^▲^ | 0.000^▲^ | 1.000 | 1.000 | 0.967 | 0.849 | 0.760 | 0.434 | NA | 1.000 | 0.809 | 0.200 | 0.247 | 0.972 | 0.026^▲^ | 0.067 |
|  | 25 | 0.000^▲^ | 0.000^▲^ | 0.000^▲^ | 0.004^▲^ | 1.000 | 1.000 | 0.821 | 0.684 | 1.000 | 0.998 | 1.000 | NA | 1.000 | 0.315 | 0.324 | 0.837 | 0.104 | 0.171 |
|  | 30 | 0.000^▲^ | 0.000^▲^ | 0.000^▲^ | 0.000^▲^ | 0.922 | 0.897 | 0.240 | 0.095 | 1.000 | 1.000 | 0.809 | 1.000 | NA | 0.024^▲^ | 0.023^▲^ | 0.158 | 0.007^▲^ | 0.010^▲^ |
|  | 35 | 0.000^▲^ | 0.000^▲^ | 0.000^▲^ | 0.002^▲^ | 0.677 | 0.752 | 1.000 | 0.999 | 0.005^▲^ | 0.005^▲^ | 0.200 | 0.315 | 0.024^▲^ | NA | 1.000 | 0.562 | 0.111 | 0.979 |
|  | 40 | 0.000^▲^ | 0.000^▲^ | 0.000^▲^ | 0.003^▲^ | 0.704 | 0.776 | 1.000 | 0.999 | 0.004^▲^ | 0.004^▲^ | 0.247 | 0.324 | 0.023^▲^ | 1.000 | NA | 0.753 | 0.747 | 1.000 |
|  | 45 | 0.000^▲^ | 0.000^▲^ | 0.000^▲^ | 0.000^▲^ | 0.999 | 1.000 | 1.000 | 1.000 | 0.060 | 0.035^▲^ | 0.972 | 0.837 | 0.158 | 0.562 | 0.753 | NA | 0.019^▲^ | 0.161 |
|  | 50 | 0.000^▲^ | 0.000^▲^ | 0.000^▲^ | 0.021^▲^ | 0.229 | 0.279 | 0.955 | 0.382 | 0.001^▲^ | 0.001^▲^ | 0.026^▲^ | 0.104 | 0.007^▲^ | 0.111 | 0.747 | 0.019^▲^ | NA | 0.999 |
|  | 55 | 0.000^▲^ | 0.000^▲^ | 0.000^▲^ | 0.010^▲^ | 0.403 | 0.475 | 0.998 | 0.848 | 0.001^▲^ | 0.002^▲^ | 0.067 | 0.171 | 0.010^▲^ | 0.979 | 1.000 | 0.161 | 0.999 | NA |
| ROI5 CT_mean_ | -30 | NA | 0.997 | 0.998 | 1.000 | 0.369 | 0.242 | 0.382 | 0.260 | 0.407 | 0.785 | 0.997 | 1.000 | 0.490 | 1.000 | 0.991 | 1.000 | 0.326 | 0.054 |
|  | -25 | 0.997 | NA | 1.000 | 1.000 | 0.001^▲^ | 0.000^▲^ | 0.000^▲^ | 0.000^▲^ | 0.000^▲^ | 0.252 | 1.000 | 0.947 | 0.000^▲^ | 0.351 | 0.017^▲^ | 0.189 | 0.188 | 0.001^▲^ |
|  | -20 | 0.998 | 1.000 | NA | 1.000 | 0.001^▲^ | 0.000^▲^ | 0.000^▲^ | 0.000^▲^ | 0.000^▲^ | 0.290 | 1.000 | 0.985 | 0.000^▲^ | 0.449 | 0.018^▲^ | 0.219 | 0.188 | 0.001^▲^ |
|  | -15 | 1.000 | 1.000 | 1.000 | NA | 0.027^▲^ | 0.007^▲^ | 0.026^▲^ | 0.008^▲^ | 0.032^▲^ | 0.565 | 1.000 | 1.000 | 0.000^▲^ | 0.965 | 0.062 | 0.639 | 0.193 | 0.001^▲^ |
|  | -10 | 0.369 | 0.001^▲^ | 0.001^▲^ | 0.027^▲^ | NA | 0.997 | 1.000 | 1.000 | 1.000 | 0.721 | 0.069 | 0.000^▲^ | 0.000^▲^ | 0.000^▲^ | 0.000^▲^ | 0.000^▲^ | 1.000 | 0.257 |
|  | -5 | 0.242 | 0.000^▲^ | 0.000^▲^ | 0.007^▲^ | 0.997 | NA | 0.794 | 1.000 | 0.497 | 0.117 | 0.015^▲^ | 0.000^▲^ | 0.000^▲^ | 0.000^▲^ | 0.000^▲^ | 0.000^▲^ | 1.000 | 0.425 |
|  | 0 | 0.382 | 0.000^▲^ | 0.000^▲^ | 0.026^▲^ | 1.000 | 0.794 | NA | 0.954 | 1.000 | 0.647 | 0.063 | 0.000^▲^ | 0.000^▲^ | 0.000^▲^ | 0.000^▲^ | 0.001^▲^ | 1.000 | 0.165 |
|  | 5 | 0.260 | 0.000^▲^ | 0.000^▲^ | 0.008^▲^ | 1.000 | 1.000 | 0.954 | NA | 0.794 | 0.166 | 0.018^▲^ | 0.000^▲^ | 0.000^▲^ | 0.000^▲^ | 0.000^▲^ | 0.000^▲^ | 1.000 | 0.387 |
|  | 10 | 0.407 | 0.000^▲^ | 0.000^▲^ | 0.032^▲^ | 1.000 | 0.497 | 1.000 | 0.794 | NA | 0.726 | 0.077 | 0.000^▲^ | 0.000^▲^ | 0.000^▲^ | 0.000^▲^ | 0.001^▲^ | 0.999 | 0.134 |
|  | 15 | 0.785 | 0.252 | 0.290 | 0.565 | 0.721 | 0.117 | 0.647 | 0.166 | 0.726 | NA | 0.859 | 0.055 | 0.000^▲^ | 0.012^▲^ | 0.001^▲^ | 0.010^▲^ | 0.874 | 0.022^▲^ |
|  | 20 | 0.997 | 1.000 | 1.000 | 1.000 | 0.069 | 0.015^▲^ | 0.063 | 0.018^▲^ | 0.077 | 0.859 | NA | 0.998 | 0.000^▲^ | 0.746 | 0.026^▲^ | 0.357 | 0.314 | 0.002^▲^ |
|  | 25 | 1.000 | 0.947 | 0.985 | 1.000 | 0.000^▲^ | 0.000^▲^ | 0.000^▲^ | 0.000^▲^ | 0.000^▲^ | 0.055 | 0.998 | NA | 0.000^▲^ | 0.975 | 0.057 | 0.621 | 0.090 | 0.000^▲^ |
|  | 30 | 0.490 | 0.000^▲^ | 0.000^▲^ | 0.000^▲^ | 0.000^▲^ | 0.000^▲^ | 0.000^▲^ | 0.000^▲^ | 0.000^▲^ | 0.000^▲^ | 0.000^▲^ | 0.000^▲^ | NA | 0.000^▲^ | 0.717 | 0.026^▲^ | 0.000^▲^ | 0.000^▲^ |
|  | 35 | 1.000 | 0.351 | 0.449 | 0.965 | 0.000^▲^ | 0.000^▲^ | 0.000^▲^ | 0.000^▲^ | 0.000^▲^ | 0.012^▲^ | 0.746 | 0.975 | 0.000^▲^ | NA | 0.336 | 0.999 | 0.032^▲^ | 0.000^▲^ |
|  | 40 | 0.991 | 0.017^▲^ | 0.018^▲^ | 0.062 | 0.000^▲^ | 0.000^▲^ | 0.000^▲^ | 0.000^▲^ | 0.000^▲^ | 0.001^▲^ | 0.026^▲^ | 0.057 | 0.717 | 0.336 | NA | 0.948 | 0.001^▲^ | 0.000^▲^ |
|  | 45 | 1.000 | 0.189 | 0.219 | 0.639 | 0.000^▲^ | 0.000^▲^ | 0.001^▲^ | 0.000^▲^ | 0.001^▲^ | 0.010^▲^ | 0.357 | 0.621 | 0.026^▲^ | 0.999 | 0.948 | NA | 0.012^▲^ | 0.000^▲^ |
|  | 50 | 0.326 | 0.188 | 0.188 | 0.193 | 1.000 | 1.000 | 1.000 | 1.000 | 0.999 | 0.874 | 0.314 | 0.090 | 0.000^▲^ | 0.032^▲^ | 0.001^▲^ | 0.012^▲^ | NA | 0.992 |
|  | 55 | 0.054 | 0.001^▲^ | 0.001^▲^ | 0.001^▲^ | 0.257 | 0.425 | 0.165 | 0.387 | 0.134 | 0.022^▲^ | 0.002^▲^ | 0.000^▲^ | 0.000^▲^ | 0.000^▲^ | 0.000^▲^ | 0.000^▲^ | 0.992 | NA |
| ROI6 CT_mean_ | -30 | NA | 1.000 | 1.000 | 1.000 | 0.164 | 0.059 | 0.032^▲^ | 0.030^▲^ | 0.138 | 0.039^▲^ | 0.047^▲^ | 0.063 | 0.427 | 0.290 | 0.002^▲^ | 0.000^▲^ | 0.000^▲^ | 0.000^▲^ |
|  | -25 | 1.000 | NA | 0.978 | 1.000 | 0.303 | 0.093 | 0.037^▲^ | 0.037^▲^ | 0.231 | 0.052 | 0.063 | 0.087 | 0.288 | 0.062 | 0.000^▲^ | 0.000^▲^ | 0.000^▲^ | 0.000^▲^ |
|  | -20 | 1.000 | 0.978 | NA | 0.995 | 0.181 | 0.131 | 0.097 | 0.095 | 0.213 | 0.110 | 0.123 | 0.144 | 0.882 | 0.999 | 0.373 | 0.050 | 0.149 | 0.160 |
|  | -15 | 1.000 | 1.000 | 0.995 | NA | 0.036^▲^ | 0.000^▲^ | 0.000^▲^ | 0.000^▲^ | 0.000^▲^ | 0.000^▲^ | 0.000^▲^ | 0.000^▲^ | 0.354 | 0.058 | 0.000^▲^ | 0.000^▲^ | 0.000^▲^ | 0.000^▲^ |
|  | -10 | 0.164 | 0.303 | 0.181 | 0.036^▲^ | NA | 1.000 | 1.000 | 1.000 | 1.000 | 1.000 | 1.000 | 1.000 | 0.048^▲^ | 0.000^▲^ | 0.000^▲^ | 0.000^▲^ | 0.000^▲^ | 0.000^▲^ |
|  | -5 | 0.059 | 0.093 | 0.131 | 0.000^▲^ | 1.000 | NA | 1.000 | 1.000 | 1.000 | 1.000 | 1.000 | 1.000 | 0.044^▲^ | 0.000^▲^ | 0.000^▲^ | 0.000^▲^ | 0.000^▲^ | 0.000^▲^ |
|  | 0 | 0.032^▲^ | 0.037^▲^ | 0.097 | 0.000^▲^ | 1.000 | 1.000 | NA | 1.000 | 0.860 | 1.000 | 1.000 | 0.979 | 0.039^▲^ | 0.000^▲^ | 0.000^▲^ | 0.000^▲^ | 0.000^▲^ | 0.000^▲^ |
|  | 5 | 0.030^▲^ | 0.037^▲^ | 0.095 | 0.000^▲^ | 1.000 | 1.000 | 1.000 | NA | 0.938 | 1.000 | 1.000 | 0.997 | 0.038^▲^ | 0.000^▲^ | 0.000^▲^ | 0.000^▲^ | 0.000^▲^ | 0.000^▲^ |
|  | 10 | 0.138 | 0.231 | 0.213 | 0.000^▲^ | 1.000 | 1.000 | 0.860 | 0.938 | NA | 0.991 | 0.998 | 1.000 | 0.062 | 0.000^▲^ | 0.000^▲^ | 0.000^▲^ | 0.000^▲^ | 0.000^▲^ |
|  | 15 | 0.039^▲^ | 0.052 | 0.110 | 0.000^▲^ | 1.000 | 1.000 | 1.000 | 1.000 | 0.991 | NA | 1.000 | 1.000 | 0.041^▲^ | 0.000^▲^ | 0.000^▲^ | 0.000^▲^ | 0.000^▲^ | 0.000^▲^ |
|  | 20 | 0.047^▲^ | 0.063 | 0.123 | 0.000^▲^ | 1.000 | 1.000 | 1.000 | 1.000 | 0.998 | 1.000 | NA | 1.000 | 0.044^▲^ | 0.000^▲^ | 0.000^▲^ | 0.000^▲^ | 0.000^▲^ | 0.000^▲^ |
|  | 25 | 0.063 | 0.087 | 0.144 | 0.000^▲^ | 1.000 | 1.000 | 0.979 | 0.997 | 1.000 | 1.000 | 1.000 | NA | 0.050^▲^ | 0.000^▲^ | 0.000^▲^ | 0.000^▲^ | 0.000^▲^ | 0.000^▲^ |
|  | 30 | 0.427 | 0.288 | 0.882 | 0.354 | 0.048^▲^ | 0.044^▲^ | 0.039^▲^ | 0.038^▲^ | 0.062 | 0.041^▲^ | 0.044^▲^ | 0.050^▲^ | NA | 0.989 | 1.000 | 1.000 | 1.000 | 1.000 |
|  | 35 | 0.290 | 0.062 | 0.999 | 0.058 | 0.000^▲^ | 0.000^▲^ | 0.000^▲^ | 0.000^▲^ | 0.000^▲^ | 0.000^▲^ | 0.000^▲^ | 0.000^▲^ | 0.989 | NA | 0.372 | 0.007^▲^ | 0.067 | 0.075 |
|  | 40 | 0.002^▲^ | 0.000^▲^ | 0.373 | 0.000^▲^ | 0.000^▲^ | 0.000^▲^ | 0.000^▲^ | 0.000^▲^ | 0.000^▲^ | 0.000^▲^ | 0.000^▲^ | 0.000^▲^ | 1.000 | 0.372 | NA | 0.059 | 0.848 | 0.887 |
|  | 45 | 0.000^▲^ | 0.000^▲^ | 0.050 | 0.000^▲^ | 0.000^▲^ | 0.000^▲^ | 0.000^▲^ | 0.000^▲^ | 0.000^▲^ | 0.000^▲^ | 0.000^▲^ | 0.000^▲^ | 1.000 | 0.007^▲^ | 0.059 | NA | 0.917 | 0.834 |
|  | 50 | 0.000^▲^ | 0.000^▲^ | 0.149 | 0.000^▲^ | 0.000^▲^ | 0.000^▲^ | 0.000^▲^ | 0.000^▲^ | 0.000^▲^ | 0.000^▲^ | 0.000^▲^ | 0.000^▲^ | 1.000 | 0.067 | 0.848 | 0.917 | NA | 1.000 |
|  | 55 | 0.000^▲^ | 0.000^▲^ | 0.160 | 0.000^▲^ | 0.000^▲^ | 0.000^▲^ | 0.000^▲^ | 0.000^▲^ | 0.000^▲^ | 0.000^▲^ | 0.000^▲^ | 0.000^▲^ | 1.000 | 0.075 | 0.887 | 0.834 | 1.000 | NA |
| ROI1 image noise | -30 | NA | 1.000 | 0.279 | 1.000 | 1.000 | 0.799 | 0.989 | 1.000 | 0.040 | 1.000 | 0.985 | 0.647 | 0.995 | 0.001^▲^ | 0.000^▲^ | 0.001^▲^ | 0.002^▲^ | 0.000^▲^ |
|  | -25 | 1.000 | NA | 0.232 | 1.000 | 1.000 | 0.995 | 1.000 | 1.000 | 0.083 | 1.000 | 0.871 | 0.442 | 0.877 | 0.020^▲^ | 0.009^▲^ | 0.019^▲^ | 0.007^▲^ | 0.005^▲^ |
|  | -20 | 0.279 | 0.232 | NA | 0.881 | 0.814 | 0.167 | 0.162 | 0.984 | 1.000 | 0.796 | 1.000 | 1.000 | 0.836 | 0.959 | 0.481 | 0.956 | 0.682 | 0.459 |
|  | -15 | 1.000 | 1.000 | 0.881 | NA | 1.000 | 0.952 | 1.000 | 1.000 | 0.658 | 1.000 | 0.999 | 0.957 | 1.000 | 0.321 | 0.161 | 0.315 | 0.163 | 0.131 |
|  | -10 | 1.000 | 1.000 | 0.814 | 1.000 | NA | 0.918 | 1.000 | 1.000 | 0.524 | 1.000 | 0.999 | 0.933 | 1.000 | 0.197 | 0.084 | 0.193 | 0.088 | 0.064 |
|  | -5 | 0.799 | 0.995 | 0.167 | 0.952 | 0.918 | NA | 1.000 | 1.000 | 0.101 | 0.844 | 0.475 | 0.231 | 0.511 | 0.050^▲^ | 0.031^▲^ | 0.048 | 0.024 | 0.023 |
|  | 0 | 0.989 | 1.000 | 0.162 | 1.000 | 1.000 | 1.000 | NA | 1.000 | 0.061 | 0.995 | 0.752 | 0.318 | 0.729 | 0.017^▲^ | 0.008^▲^ | 0.016 | 0.006 | 0.005 |
|  | 5 | 1.000 | 1.000 | 0.984 | 1.000 | 1.000 | 1.000 | 1.000 | NA | 0.952 | 1.000 | 0.999 | 0.992 | 1.000 | 0.852 | 0.734 | 0.847 | 0.709 | 0.687 |
|  | 10 | 0.040^▲^ | 0.083 | 1.000 | 0.658 | 0.524 | 0.101 | 0.061 | 0.952 | NA | 0.455 | 0.999 | 1.000 | 0.312 | 0.999 | 0.616 | 0.998 | 0.856 | 0.627 |
|  | 15 | 1.000 | 1.000 | 0.796 | 1.000 | 1.000 | 0.844 | 0.995 | 1.000 | 0.455 | NA | 0.999 | 0.937 | 1.000 | 0.130 | 0.047^▲^ | 0.127 | 0.054 | 0.034^▲^ |
|  | 20 | 0.985 | 0.871 | 1.000 | 0.999 | 0.999 | 0.475 | 0.752 | 0.999 | 0.999 | 0.999 | NA | 1.000 | 1.000 | 0.916 | 0.637 | 0.911 | 0.675 | 0.574 |
|  | 25 | 0.647 | 0.442 | 1.000 | 0.957 | 0.933 | 0.231 | 0.318 | 0.992 | 1.000 | 0.937 | 1.000 | NA | 0.978 | 0.980 | 0.713 | 0.978 | 0.790 | 0.657 |
|  | 30 | 0.995 | 0.877 | 0.836 | 1.000 | 1.000 | 0.511 | 0.729 | 1.000 | 0.312 | 1.000 | 1.000 | 0.978 | NA | 0.014^▲^ | 0.002^▲^ | 0.016^▲^ | 0.017^▲^ | 0.001^▲^ |
|  | 35 | 0.001^▲^ | 0.020^▲^ | 0.959 | 0.321 | 0.197 | 0.050^▲^ | 0.017^▲^ | 0.852 | 0.999 | 0.130 | 0.916 | 0.980 | 0.014^▲^ | NA | 0.979 | 1.000 | 0.997 | 0.972 |
|  | 40 | 0.000^▲^ | 0.009^▲^ | 0.481 | 0.161 | 0.084 | 0.031^▲^ | 0.008^▲^ | 0.734 | 0.616 | 0.047^▲^ | 0.637 | 0.713 | 0.002^▲^ | 0.979 | NA | 0.995 | 1.000 | 1.000 |
|  | 45 | 0.001^▲^ | 0.019^▲^ | 0.956 | 0.315 | 0.193 | 0.048^▲^ | 0.016^▲^ | 0.847 | 0.998 | 0.127 | 0.911 | 0.978 | 0.016^▲^ | 1.000 | 0.995 | NA | 0.999 | 0.987 |
|  | 50 | 0.002^▲^ | 0.007^▲^ | 0.682 | 0.163 | 0.088 | 0.024^▲^ | 0.006^▲^ | 0.709 | 0.856 | 0.054 | 0.675 | 0.790 | 0.017^▲^ | 0.997 | 1.000 | 0.999 | NA | 1.000 |
|  | 55 | 0.000^▲^ | 0.005^▲^ | 0.459 | 0.131 | 0.064 | 0.023^▲^ | 0.005^▲^ | 0.687 | 0.627 | 0.034^▲^ | 0.574 | 0.657 | 0.001^▲^ | 0.972 | 1.000 | 0.987 | 1.000 | NA |
| ROI2 image noise | -30 | NA | 1.000 | 1.000 | 0.498 | 0.003^▲^ | 0.995 | 1.000 | 0.078 | 0.325 | 0.027^▲^ | 0.382 | 0.392 | 0.000^▲^ | 0.000^▲^ | 0.003^▲^ | 0.806 | 0.935 | 1.000 |
|  | -25 | 1.000 | NA | 1.000 | 0.830 | 0.054 | 1.000 | 1.000 | 0.289 | 0.904 | 0.191 | 0.978 | 0.539 | 0.011^▲^ | 0.015^▲^ | 0.066 | 0.928 | 0.977 | 1.000 |
|  | -20 | 1.000 | 1.000 | NA | 0.493 | 0.003^▲^ | 0.995 | 1.000 | 0.076 | 0.342 | 0.027^▲^ | 0.426 | 0.467 | 0.000^▲^ | 0.000^▲^ | 0.007^▲^ | 0.900 | 0.977 | 1.000 |
|  | -15 | 0.498 | 0.830 | 0.493 | NA | 0.999 | 0.920 | 0.624 | 1.000 | 1.000 | 1.000 | 0.998 | 0.047^▲^ | 0.003^▲^ | 0.004^▲^ | 0.009^▲^ | 0.133 | 0.176 | 0.384 |
|  | -10 | 0.003^▲^ | 0.054 | 0.003^▲^ | 0.999 | NA | 0.042^▲^ | 0.081 | 1.000 | 0.566 | 1.000 | 0.159 | 0.000^▲^ | 0.000^▲^ | 0.000^▲^ | 0.000^▲^ | 0.000^▲^ | 0.000^▲^ | 0.001^▲^ |
|  | -5 | 0.995 | 1.000 | 0.995 | 0.920 | 0.042^▲^ | NA | 0.994 | 0.361 | 0.974 | 0.218 | 0.999 | 0.154 | 0.000^▲^ | 0.000^▲^ | 0.002^▲^ | 0.365 | 0.509 | 0.945 |
|  | 0 | 1.000 | 1.000 | 1.000 | 0.624 | 0.081 | 0.994 | NA | 0.220 | 0.715 | 0.175 | 0.839 | 0.996 | 0.420 | 0.470 | 0.810 | 1.000 | 1.000 | 1.000 |
|  | 5 | 0.078 | 0.289 | 0.076 | 1.000 | 1.000 | 0.361 | 0.220 | NA | 0.961 | 1.000 | 0.725 | 0.004^▲^ | 0.000^▲^ | 0.000^▲^ | 0.000^▲^ | 0.011^▲^ | 0.017^▲^ | 0.051 |
|  | 10 | 0.325 | 0.904 | 0.342 | 1.000 | 0.566 | 0.974 | 0.715 | 0.961 | NA | 0.926 | 1.000 | 0.019^▲^ | 0.000^▲^ | 0.000^▲^ | 0.000^▲^ | 0.032^▲^ | 0.049^▲^ | 0.186 |
|  | 15 | 0.027^▲^ | 0.191 | 0.027^▲^ | 1.000 | 1.000 | 0.218 | 0.175 | 1.000 | 0.926 | NA | 0.569 | 0.002^▲^ | 0.000^▲^ | 0.000^▲^ | 0.000^▲^ | 0.003^▲^ | 0.004^▲^ | 0.016^▲^ |
|  | 20 | 0.382 | 0.978 | 0.426 | 0.998 | 0.159 | 0.999 | 0.839 | 0.725 | 1.000 | 0.569 | NA | 0.028^▲^ | 0.000^▲^ | 0.000^▲^ | 0.000^▲^ | 0.029^▲^ | 0.045^▲^ | 0.186 |
|  | 25 | 0.392 | 0.539 | 0.467 | 0.047^▲^ | 0.000^▲^ | 0.154 | 0.996 | 0.004^▲^ | 0.019^▲^ | 0.002^▲^ | 0.028^▲^ | NA | 0.884 | 0.926 | 1.000 | 0.996 | 0.968 | 0.531 |
|  | 30 | 0.000^▲^ | 0.011^▲^ | 0.000^▲^ | 0.003^▲^ | 0.000^▲^ | 0.000^▲^ | 0.420 | 0.000^▲^ | 0.000^▲^ | 0.000^▲^ | 0.000^▲^ | 0.884 | NA | 1.000 | 0.986 | 0.024^▲^ | 0.007^▲^ | 0.000^▲^ |
|  | 35 | 0.000^▲^ | 0.015^▲^ | 0.000^▲^ | 0.004^▲^ | 0.000^▲^ | 0.000^▲^ | 0.470 | 0.000^▲^ | 0.000^▲^ | 0.000^▲^ | 0.000^▲^ | 0.926 | 1.000 | NA | 0.997 | 0.028^▲^ | 0.008^▲^ | 0.000^▲^ |
|  | 40 | 0.003^▲^ | 0.066 | 0.007^▲^ | 0.009^▲^ | 0.000^▲^ | 0.002^▲^ | 0.810 | 0.000^▲^ | 0.000^▲^ | 0.000^▲^ | 0.000^▲^ | 1.000 | 0.986 | 0.997 | NA | 0.353 | 0.166 | 0.006^▲^ |
|  | 45 | 0.806 | 0.928 | 0.900 | 0.133 | 0.000^▲^ | 0.365 | 1.000 | 0.011^▲^ | 0.032^▲^ | 0.003^▲^ | 0.029^▲^ | 0.996 | 0.024^▲^ | 0.028^▲^ | 0.353 | NA | 1.000 | 0.946 |
|  | 50 | 0.935 | 0.977 | 0.977 | 0.176 | 0.000^▲^ | 0.509 | 1.000 | 0.017^▲^ | 0.049^▲^ | 0.004^▲^ | 0.045^▲^ | 0.968 | 0.007^▲^ | 0.008^▲^ | 0.166 | 1.000 | NA | 0.993 |
|  | 55 | 1.000 | 1.000 | 1.000 | 0.384 | 0.001^▲^ | 0.945 | 1.000 | 0.051 | 0.186 | 0.016^▲^ | 0.186 | 0.531 | 0.000^▲^ | 0.000^▲^ | 0.006^▲^ | 0.946 | 0.993 | NA |
| ROI3 image noise | -30 | NA | 0.999 | 0.999 | 1.000 | 1.000 | 1.000 | 1.000 | 0.766 | 1.000 | 1.000 | 1.000 | 0.456 | 0.068 | 0.026^▲^ | 0.117 | 0.300 | 0.395 | 0.567 |
|  | -25 | 0.999 | NA | 1.000 | 0.991 | 0.958 | 0.990 | 1.000 | 0.003^▲^ | 1.000 | 0.990 | 1.000 | 0.407 | 0.002^▲^ | 0.000^▲^ | 0.006^▲^ | 0.062 | 0.154 | 0.393 |
|  | -20 | 0.999 | 1.000 | NA | 0.988 | 0.956 | 0.986 | 1.000 | 0.010^▲^ | 1.000 | 0.986 | 1.000 | 0.709 | 0.021^▲^ | 0.004^▲^ | 0.058 | 0.296 | 0.505 | 0.804 |
|  | -15 | 1.000 | 0.991 | 0.988 | NA | 1.000 | 1.000 | 1.000 | 0.011^▲^ | 1.000 | 1.000 | 1.000 | 0.034^▲^ | 0.000^▲^ | 0.000^▲^ | 0.000^▲^ | 0.000^▲^ | 0.001^▲^ | 0.003^▲^ |
|  | -10 | 1.000 | 0.958 | 0.956 | 1.000 | NA | 1.000 | 0.999 | 0.066 | 0.996 | 1.000 | 0.999 | 0.029^▲^ | 0.000^▲^ | 0.000^▲^ | 0.000^▲^ | 0.002^▲^ | 0.004^▲^ | 0.013^▲^ |
|  | -5 | 1.000 | 0.990 | 0.986 | 1.000 | 1.000 | NA | 1.000 | 0.096 | 0.999 | 1.000 | 1.000 | 0.061 | 0.001^▲^ | 0.000^▲^ | 0.001^▲^ | 0.010^▲^ | 0.018^▲^ | 0.051 |
|  | 0 | 1.000 | 1.000 | 1.000 | 1.000 | 0.999 | 1.000 | NA | 0.039^▲^ | 1.000 | 1.000 | 1.000 | 0.531 | 0.019^▲^ | 0.004^▲^ | 0.045^▲^ | 0.213 | 0.357 | 0.621 |
|  | 5 | 0.766 | 0.003^▲^ | 0.010^▲^ | 0.011^▲^ | 0.066 | 0.096 | 0.039^▲^ | NA | 0.046^▲^ | 0.337 | 0.061 | 0.000^▲^ | 0.000^▲^ | 0.000^▲^ | 0.000^▲^ | 0.000^▲^ | 0.000^▲^ | 0.000^▲^ |
|  | 10 | 1.000 | 1.000 | 1.000 | 1.000 | 0.996 | 0.999 | 1.000 | 0.046^▲^ | NA | 0.999 | 1.000 | 0.733 | 0.047^▲^ | 0.012^▲^ | 0.111 | 0.402 | 0.590 | 0.837 |
|  | 15 | 1.000 | 0.990 | 0.986 | 1.000 | 1.000 | 1.000 | 1.000 | 0.337 | 0.999 | NA | 1.000 | 0.119 | 0.005^▲^ | 0.001^▲^ | 0.008^▲^ | 0.044^▲^ | 0.070 | 0.146 |
|  | 20 | 1.000 | 1.000 | 1.000 | 1.000 | 0.999 | 1.000 | 1.000 | 0.061 | 1.000 | 1.000 | NA | 0.618 | 0.033^▲^ | 0.008^▲^ | 0.077 | 0.303 | 0.466 | 0.725 |
|  | 25 | 0.456 | 0.407 | 0.709 | 0.034^▲^ | 0.029^▲^ | 0.061 | 0.531 | 0.000^▲^ | 0.733 | 0.119 | 0.618 | NA | 0.604 | 0.138 | 0.967 | 1.000 | 1.000 | 1.000 |
|  | 30 | 0.068 | 0.002^▲^ | 0.021^▲^ | 0.000^▲^ | 0.000^▲^ | 0.001^▲^ | 0.019^▲^ | 0.000^▲^ | 0.047^▲^ | 0.005^▲^ | 0.033^▲^ | 0.604 | NA | 0.428 | 0.997 | 0.001^▲^ | 0.027^▲^ | 0.001^▲^ |
|  | 35 | 0.026^▲^ | 0.000^▲^ | 0.004^▲^ | 0.000^▲^ | 0.000^▲^ | 0.000^▲^ | 0.004^▲^ | 0.000^▲^ | 0.012^▲^ | 0.001^▲^ | 0.008^▲^ | 0.138 | 0.428 | NA | 0.279 | 0.000^▲^ | 0.001^▲^ | 0.000^▲^ |
|  | 40 | 0.117 | 0.006^▲^ | 0.058 | 0.000^▲^ | 0.000^▲^ | 0.001^▲^ | 0.045^▲^ | 0.000^▲^ | 0.111 | 0.008^▲^ | 0.077 | 0.967 | 0.997 | 0.279 | NA | 0.547 | 0.528 | 0.091 |
|  | 45 | 0.300 | 0.062 | 0.296 | 0.000^▲^ | 0.002^▲^ | 0.010^▲^ | 0.213 | 0.000^▲^ | 0.402 | 0.044^▲^ | 0.303 | 1.000 | 0.001^▲^ | 0.000^▲^ | 0.547 | NA | 1.000 | 0.792 |
|  | 50 | 0.395 | 0.154 | 0.505 | 0.001^▲^ | 0.004^▲^ | 0.018^▲^ | 0.357 | 0.000^▲^ | 0.590 | 0.070 | 0.466 | 1.000 | 0.027^▲^ | 0.001^▲^ | 0.528 | 1.000 | NA | 0.999 |
|  | 55 | 0.567 | 0.393 | 0.804 | 0.003^▲^ | 0.013^▲^ | 0.051 | 0.621 | 0.000^▲^ | 0.837 | 0.146 | 0.725 | 1.000 | 0.001^▲^ | 0.000^▲^ | 0.091 | 0.792 | 0.999 | NA |
| ROI4 image noise | -30 | NA | 1.000 | 0.852 | 0.731 | 0.007^▲^ | 0.057 | 0.285 | 0.000^▲^ | 0.000^▲^ | 0.000^▲^ | 0.001^▲^ | 0.000^▲^ | 0.000^▲^ | 0.005^▲^ | 0.018^▲^ | 0.094 | 0.878 | 0.175 |
|  | -25 | 1.000 | NA | 0.998 | 0.992 | 0.068 | 0.347 | 0.885 | 0.009^▲^ | 0.000^▲^ | 0.000^▲^ | 0.004^▲^ | 0.000^▲^ | 0.000^▲^ | 0.086 | 0.209 | 0.507 | 1.000 | 0.813 |
|  | -20 | 0.852 | 0.998 | NA | 1.000 | 0.438 | 0.943 | 1.000 | 0.089 | 0.002^▲^ | 0.000^▲^ | 0.027^▲^ | 0.001^▲^ | 0.000^▲^ | 0.557 | 0.853 | 0.992 | 1.000 | 1.000 |
|  | -15 | 0.731 | 0.992 | 1.000 | NA | 0.462 | 0.961 | 1.000 | 0.084 | 0.002^▲^ | 0.000^▲^ | 0.029^▲^ | 0.001^▲^ | 0.000^▲^ | 0.584 | 0.882 | 0.996 | 0.998 | 1.000 |
|  | -10 | 0.007^▲^ | 0.068 | 0.438 | 0.462 | NA | 0.999 | 0.476 | 1.000 | 0.370 | 0.003^▲^ | 0.742 | 0.037^▲^ | 0.003^▲^ | 1.000 | 0.999 | 0.978 | 0.033^▲^ | 0.395 |
|  | -5 | 0.057 | 0.347 | 0.943 | 0.961 | 0.999 | NA | 0.983 | 0.853 | 0.054 | 0.001^▲^ | 0.273 | 0.008^▲^ | 0.000^▲^ | 1.000 | 1.000 | 1.000 | 0.222 | 0.971 |
|  | 0 | 0.285 | 0.885 | 1.000 | 1.000 | 0.476 | 0.983 | NA | 0.049^▲^ | 0.001^▲^ | 0.000^▲^ | 0.032^▲^ | 0.001^▲^ | 0.000^▲^ | 0.589 | 0.921 | 0.999 | 0.785 | 1.000 |
|  | 5 | 0.000^▲^ | 0.009^▲^ | 0.089 | 0.084 | 1.000 | 0.853 | 0.049^▲^ | NA | 0.582 | 0.005^▲^ | 0.917 | 0.069 | 0.001^▲^ | 0.989 | 0.793 | 0.541 | 0.001^▲^ | 0.024^▲^ |
|  | 10 | 0.000^▲^ | 0.000^▲^ | 0.002^▲^ | 0.002^▲^ | 0.370 | 0.054 | 0.001^▲^ | 0.582 | NA | 0.084 | 1.000 | 0.708 | 0.309 | 0.095 | 0.027^▲^ | 0.016^▲^ | 0.000^▲^ | 0.000^▲^ |
|  | 15 | 0.000^▲^ | 0.000^▲^ | 0.000^▲^ | 0.000^▲^ | 0.003^▲^ | 0.001^▲^ | 0.000^▲^ | 0.005^▲^ | 0.084 | NA | 0.197 | 0.991 | 0.671 | 0.001^▲^ | 0.001^▲^ | 0.000^▲^ | 0.000^▲^ | 0.000^▲^ |
|  | 20 | 0.001^▲^ | 0.004^▲^ | 0.027^▲^ | 0.029^▲^ | 0.742 | 0.273 | 0.032^▲^ | 0.917 | 1.000 | 0.197 | NA | 0.887 | 0.815 | 0.452 | 0.243 | 0.147 | 0.006^▲^ | 0.030^▲^ |
|  | 25 | 0.000^▲^ | 0.000^▲^ | 0.001^▲^ | 0.001^▲^ | 0.037^▲^ | 0.008^▲^ | 0.001^▲^ | 0.069 | 0.708 | 0.991 | 0.887 | NA | 1.000 | 0.015^▲^ | 0.007^▲^ | 0.004^▲^ | 0.001^▲^ | 0.001^▲^ |
|  | 30 | 0.000^▲^ | 0.000^▲^ | 0.000^▲^ | 0.000^▲^ | 0.003^▲^ | 0.000^▲^ | 0.000^▲^ | 0.001^▲^ | 0.309 | 0.671 | 0.815 | 1.000 | NA | 0.000^▲^ | 0.000^▲^ | 0.000^▲^ | 0.000^▲^ | 0.000^▲^ |
|  | 35 | 0.005^▲^ | 0.086 | 0.557 | 0.584 | 1.000 | 1.000 | 0.589 | 0.989 | 0.095 | 0.001^▲^ | 0.452 | 0.015^▲^ | 0.000^▲^ | NA | 1.000 | 0.998 | 0.019^▲^ | 0.470 |
|  | 40 | 0.018^▲^ | 0.209 | 0.853 | 0.882 | 0.999 | 1.000 | 0.921 | 0.793 | 0.027^▲^ | 0.001^▲^ | 0.243 | 0.007^▲^ | 0.000^▲^ | 1.000 | NA | 1.000 | 0.063 | 0.865 |
|  | 45 | 0.094 | 0.507 | 0.992 | 0.996 | 0.978 | 1.000 | 0.999 | 0.541 | 0.016^▲^ | 0.000^▲^ | 0.147 | 0.004^▲^ | 0.000^▲^ | 0.998 | 1.000 | NA | 0.340 | 0.999 |
|  | 50 | 0.878 | 1.000 | 1.000 | 0.998 | 0.033^▲^ | 0.222 | 0.785 | 0.001^▲^ | 0.000^▲^ | 0.000^▲^ | 0.006^▲^ | 0.001^▲^ | 0.000^▲^ | 0.019^▲^ | 0.063 | 0.340 | NA | 0.493 |
|  | 55 | 0.175 | 0.813 | 1.000 | 1.000 | 0.395 | 0.971 | 1.000 | 0.024^▲^ | 0.000^▲^ | 0.000^▲^ | 0.030^▲^ | 0.001^▲^ | 0.000^▲^ | 0.470 | 0.865 | 0.999 | 0.493 | NA |
| ROI5 image noise | -30 | NA | 1.000 | 0.493 | 0.315 | 0.038^▲^ | 0.091 | 0.251 | 0.146 | 0.029^▲^ | 0.133 | 1.000 | 1.000 | 1.000 | 0.963 | 0.002^▲^ | 0.007^▲^ | 0.004^▲^ | 0.001^▲^ |
|  | -25 | 1.000 | NA | 0.055 | 0.021^▲^ | 0.001^▲^ | 0.011^▲^ | 0.031^▲^ | 0.011^▲^ | 0.001^▲^ | 0.021^▲^ | 0.986 | 1.000 | 1.000 | 0.996 | 0.002^▲^ | 0.011^▲^ | 0.006^▲^ | 0.001^▲^ |
|  | -20 | 0.493 | 0.055 | NA | 1.000 | 0.493 | 0.901 | 1.000 | 0.993 | 0.562 | 0.956 | 0.627 | 0.027^▲^ | 0.102 | 0.017^▲^ | 0.000^▲^ | 0.000^▲^ | 0.001^▲^ | 0.000^▲^ |
|  | -15 | 0.315 | 0.021^▲^ | 1.000 | NA | 0.703 | 0.969 | 1.000 | 1.000 | 0.739 | 0.990 | 0.368 | 0.008^▲^ | 0.045^▲^ | 0.009^▲^ | 0.000^▲^ | 0.000^▲^ | 0.001^▲^ | 0.000^▲^ |
|  | -10 | 0.038^▲^ | 0.001^▲^ | 0.493 | 0.703 | NA | 1.000 | 1.000 | 1.000 | 1.000 | 1.000 | 0.017 | 0.000^▲^ | 0.002^▲^ | 0.001^▲^ | 0.000^▲^ | 0.000^▲^ | 0.001^▲^ | 0.000^▲^ |
|  | -5 | 0.091 | 0.011^▲^ | 0.901 | 0.969 | 1.000 | NA | 1.000 | 1.000 | 1.000 | 1.000 | 0.116 | 0.010^▲^ | 0.017^▲^ | 0.003^▲^ | 0.000^▲^ | 0.000^▲^ | 0.000^▲^ | 0.000^▲^ |
|  | 0 | 0.251 | 0.031^▲^ | 1.000 | 1.000 | 1.000 | 1.000 | NA | 1.000 | 0.997 | 1.000 | 0.329 | 0.022^▲^ | 0.050 | 0.008^▲^ | 0.000^▲^ | 0.000^▲^ | 0.000^▲^ | 0.000^▲^ |
|  | 5 | 0.146 | 0.011^▲^ | 0.993 | 1.000 | 1.000 | 1.000 | 1.000 | NA | 1.000 | 1.000 | 0.169 | 0.007^▲^ | 0.020^▲^ | 0.004^▲^ | 0.000^▲^ | 0.000^▲^ | 0.000^▲^ | 0.000^▲^ |
|  | 10 | 0.029^▲^ | 0.001^▲^ | 0.562 | 0.739 | 1.000 | 1.000 | 0.997 | 1.000 | NA | 1.000 | 0.022 | 0.001^▲^ | 0.002^▲^ | 0.001^▲^ | 0.000^▲^ | 0.000^▲^ | 0.000^▲^ | 0.000^▲^ |
|  | 15 | 0.133 | 0.021^▲^ | 0.956 | 0.990 | 1.000 | 1.000 | 1.000 | 1.000 | 1.000 | NA | 0.181 | 0.019^▲^ | 0.031^▲^ | 0.005^▲^ | 0.000^▲^ | 0.000^▲^ | 0.000^▲^ | 0.000^▲^ |
|  | 20 | 1.000 | 0.986 | 0.627 | 0.368 | 0.017^▲^ | 0.116 | 0.329 | 0.169 | 0.022^▲^ | 0.181 | NA | 0.986 | 0.996 | 0.558 | 0.000^▲^ | 0.003^▲^ | 0.003^▲^ | 0.000^▲^ |
|  | 25 | 1.000 | 1.000 | 0.027^▲^ | 0.008^▲^ | 0.000^▲^ | 0.010^▲^ | 0.022^▲^ | 0.007^▲^ | 0.001^▲^ | 0.019^▲^ | 0.986 | NA | 1.000 | 0.979 | 0.001^▲^ | 0.009^▲^ | 0.006^▲^ | 0.001^▲^ |
|  | 30 | 1.000 | 1.000 | 0.102 | 0.045^▲^ | 0.002^▲^ | 0.017^▲^ | 0.050 | 0.020^▲^ | 0.002^▲^ | 0.031^▲^ | 0.996 | 1.000 | NA | 0.993 | 0.002^▲^ | 0.010^▲^ | 0.005^▲^ | 0.001^▲^ |
|  | 35 | 0.963 | 0.996 | 0.017^▲^ | 0.009^▲^ | 0.001^▲^ | 0.003^▲^ | 0.008^▲^ | 0.004^▲^ | 0.001^▲^ | 0.005^▲^ | 0.558 | 0.979 | 0.993 | NA | 0.026^▲^ | 0.058 | 0.014^▲^ | 0.003^▲^ |
|  | 40 | 0.002^▲^ | 0.002^▲^ | 0.000^▲^ | 0.000^▲^ | 0.000^▲^ | 0.000^▲^ | 0.000^▲^ | 0.000^▲^ | 0.000^▲^ | 0.000^▲^ | 0.000^▲^ | 0.001^▲^ | 0.002^▲^ | 0.026^▲^ | NA | 1.000 | 0.588 | 0.459 |
|  | 45 | 0.007^▲^ | 0.011^▲^ | 0.000^▲^ | 0.000^▲^ | 0.000^▲^ | 0.000^▲^ | 0.000^▲^ | 0.000^▲^ | 0.000^▲^ | 0.000^▲^ | 0.003^▲^ | 0.009^▲^ | 0.010^▲^ | 0.058 | 1.000 | NA | 0.871 | 0.833 |
|  | 50 | 0.004^▲^ | 0.006^▲^ | 0.001^▲^ | 0.001^▲^ | 0.001^▲^ | 0.000^▲^ | 0.000^▲^ | 0.000^▲^ | 0.000^▲^ | 0.000^▲^ | 0.003^▲^ | 0.006^▲^ | 0.005^▲^ | 0.014^▲^ | 0.588 | 0.871 | NA | 1.000 |
|  | 55 | 0.001^▲^ | 0.001^▲^ | 0.000^▲^ | 0.000^▲^ | 0.000^▲^ | 0.000^▲^ | 0.000^▲^ | 0.000^▲^ | 0.000^▲^ | 0.000^▲^ | 0.000^▲^ | 0.001^▲^ | 0.001^▲^ | 0.003^▲^ | 0.459 | 0.833 | 1.000 | NA |
| ROI6 image noise | -30 | NA | 1.000 | 0.999 | 1.000 | 0.658 | 1.000 | 1.000 | 0.996 | 0.828 | 0.996 | 0.528 | 1.000 | 1.000 | 0.999 | 0.999 | 0.840 | 0.013 | 0.001 |
|  | -25 | 1.000 | NA | 0.945 | 1.000 | 0.763 | 1.000 | 1.000 | 1.000 | 0.913 | 1.000 | 0.234 | 1.000 | 0.997 | 0.967 | 0.987 | 0.574 | 0.002^▲^ | 0.000^▲^ |
|  | -20 | 0.999 | 0.945 | NA | 0.995 | 0.071 | 0.932 | 0.824 | 0.627 | 0.140 | 0.548 | 0.920 | 1.000 | 1.000 | 1.000 | 1.000 | 0.997 | 0.032^▲^ | 0.001^▲^ |
|  | -15 | 1.000 | 1.000 | 0.995 | NA | 0.485 | 1.000 | 1.000 | 0.989 | 0.696 | 0.989 | 0.366 | 1.000 | 1.000 | 0.996 | 0.998 | 0.743 | 0.004^▲^ | 0.000^▲^ |
|  | -10 | 0.658 | 0.763 | 0.071 | 0.485 | NA | 0.999 | 0.973 | 1.000 | 1.000 | 1.000 | 0.007^▲^ | 0.673 | 0.190 | 0.161 | 0.419 | 0.040^▲^ | 0.000^▲^ | 0.000^▲^ |
|  | -5 | 1.000 | 1.000 | 0.932 | 1.000 | 0.999 | NA | 1.000 | 1.000 | 1.000 | 1.000 | 0.373 | 1.000 | 0.986 | 0.943 | 0.968 | 0.618 | 0.025^▲^ | 0.004^▲^ |
|  | 0 | 1.000 | 1.000 | 0.824 | 1.000 | 0.973 | 1.000 | NA | 1.000 | 0.997 | 1.000 | 0.166 | 1.000 | 0.966 | 0.884 | 0.952 | 0.432 | 0.002^▲^ | 0.000^▲^ |
|  | 5 | 0.996 | 1.000 | 0.627 | 0.989 | 1.000 | 1.000 | 1.000 | NA | 1.000 | 1.000 | 0.119 | 0.991 | 0.835 | 0.706 | 0.843 | 0.293 | 0.003^▲^ | 0.000^▲^ |
|  | 10 | 0.828 | 0.913 | 0.140 | 0.696 | 1.000 | 1.000 | 0.997 | 1.000 | NA | 1.000 | 0.015^▲^ | 0.820 | 0.324 | 0.261 | 0.538 | 0.069 | 0.000^▲^ | 0.000^▲^ |
|  | 15 | 0.996 | 1.000 | 0.548 | 0.989 | 1.000 | 1.000 | 1.000 | 1.000 | 1.000 | NA | 0.080 | 0.992 | 0.805 | 0.669 | 0.840 | 0.247 | 0.001^▲^ | 0.000^▲^ |
|  | 20 | 0.528 | 0.234 | 0.920 | 0.366 | 0.007^▲^ | 0.373 | 0.166 | 0.119 | 0.015^▲^ | 0.080 | NA | 0.834 | 0.853 | 0.995 | 1.000 | 1.000 | 0.816 | 0.134 |
|  | 25 | 1.000 | 1.000 | 1.000 | 1.000 | 0.673 | 1.000 | 1.000 | 0.991 | 0.820 | 0.992 | 0.834 | NA | 1.000 | 1.000 | 1.000 | 0.970 | 0.075 | 0.008^▲^ |
|  | 30 | 1.000 | 0.997 | 1.000 | 1.000 | 0.190 | 0.986 | 0.966 | 0.835 | 0.324 | 0.805 | 0.853 | 1.000 | NA | 1.000 | 1.000 | 0.986 | 0.031^▲^ | 0.001^▲^ |
|  | 35 | 0.999 | 0.967 | 1.000 | 0.996 | 0.161 | 0.943 | 0.884 | 0.706 | 0.261 | 0.669 | 0.995 | 1.000 | 1.000 | NA | 1.000 | 1.000 | 0.199 | 0.017^▲^ |
|  | 40 | 0.999 | 0.987 | 1.000 | 0.998 | 0.419 | 0.968 | 0.952 | 0.843 | 0.538 | 0.840 | 1.000 | 1.000 | 1.000 | 1.000 | NA | 1.000 | 0.717 | 0.237 |
|  | 45 | 0.840 | 0.574 | 0.997 | 0.743 | 0.040^▲^ | 0.618 | 0.432 | 0.293 | 0.069 | 0.247 | 1.000 | 0.970 | 0.986 | 1.000 | 1.000 | NA | 0.748 | 0.148 |
|  | 50 | 0.013^▲^ | 0.002^▲^ | 0.032^▲^ | 0.004^▲^ | 0.000^▲^ | 0.025^▲^ | 0.002^▲^ | 0.003^▲^ | 0.000^▲^ | 0.001^▲^ | 0.816 | 0.075 | 0.031^▲^ | 0.199 | 0.717 | 0.748 | NA | 0.979 |
|  | 55 | 0.001^▲^ | 0.000^▲^ | 0.001^▲^ | 0.000^▲^ | 0.000^▲^ | 0.004^▲^ | 0.000^▲^ | 0.000^▲^ | 0.000^▲^ | 0.000^▲^ | 0.134 | 0.008^▲^ | 0.001^▲^ | 0.017^▲^ | 0.237 | 0.148 | 0.979 | NA |

Abbreviations: CTDI_vol_, computed tomography dose index volume ; SSDE, size-specific dose estimate; DLP, dose-length product; CT_mean_ , mean CT value; NA, not applicable, ^▲^ indicates p＜0.05.
